# AML1/ETO Oncoprotein Is Directed to AML1 Binding Regions and Co-Localizes with AML1 and HEB on Its Targets

**DOI:** 10.1371/journal.pgen.1000275

**Published:** 2008-11-28

**Authors:** Alessandro Gardini, Matteo Cesaroni, Lucilla Luzi, Akiko J. Okumura, Joseph R. Biggs, Simone P. Minardi, Elisa Venturini, Dong-Er Zhang, Pier Giuseppe Pelicci, Myriam Alcalay

**Affiliations:** 1 Department of Experimental Oncology, IEO–European Institute of Oncology, Milan, Italy; 2 IFOM–FIRC Institute for Molecular Oncology Foundation, Milan, Italy; 3 Moores UCSD Cancer Center, Department of Pathology and Division of Biological Sciences, University of California San Diego, La Jolla, California, United States of America; 4 Cogentech–Consortium for Genomic Technologies, Milan, Italy; 5 Dipartimento di Medicina, Chirurgia e Odontoiatria, Università degli Studi di Milano, Milan, Italy; University of Pennsylvania, United States of America

## Abstract

A reciprocal translocation involving chromosomes 8 and 21 generates the AML1/ETO oncogenic transcription factor that initiates acute myeloid leukemia by recruiting co-repressor complexes to DNA. AML1/ETO interferes with the function of its wild-type counterpart, AML1, by directly targeting AML1 binding sites. However, transcriptional regulation determined by AML1/ETO probably relies on a more complex network, since the fusion protein has been shown to interact with a number of other transcription factors, in particular E-proteins, and may therefore target other sites on DNA. Genome-wide chromatin immunoprecipitation and expression profiling were exploited to identify AML1/ETO-dependent transcriptional regulation. AML1/ETO was found to co-localize with AML1, demonstrating that the fusion protein follows the binding pattern of the wild-type protein but does not function primarily by displacing it. The DNA binding profile of the E-protein HEB was grossly rearranged upon expression of AML1/ETO, and the fusion protein was found to co-localize with both AML1 and HEB on many of its regulated targets. Furthermore, the level of HEB protein was increased in both primary cells and cell lines expressing AML1/ETO. Our results suggest a major role for the functional interaction of AML1/ETO with AML1 and HEB in transcriptional regulation determined by the fusion protein.

## Author Summary

Acute myeloid leukemias (AML) are a group of hematologic malignancies initiated by chromosomal abnormalities that often give origin to oncogenic proteins with transcriptional regulatory functions. These aberrant transcription factors bind to specific sequences on DNA and influence the activity of adjacent genes. The result is that leukemic blasts display abnormalities in their gene expression programs, which are ultimately responsible for the malignant phenotype. In this study, genome-wide approaches were exploited not only to identify target genes, but also to discover interactions among different transcription factors, with the aim of defining disease-linked regulatory networks. We performed a detailed analysis of the DNA binding pattern of an oncogenic transcription factor, AML1/ETO, which is responsible for approximately 10–15% of AML. We identified a specific signature, which is characterized by the presence of binding regions for AML1/ETO and for other transcription factors, AML1 and HEB, and found that the DNA binding pattern of AML1 and HEB is significantly affected in cells expressing AML1/ETO. Our results, therefore, describe genes regulated by AML1/ETO and demonstrate that this oncogenic protein can significantly interfere with the function of other transcriptional regulators.

## Introduction

Chromosomal translocations generating fusion genes are the genetic hallmark of Acute Myeloid Leukemia (AML) [1]. Approximately 10–15% of AML cases carry the t(8;21) translocation, which involves the *AML1* and *ETO* genes, and express the resulting AML1/ETO fusion protein. AML1 is a DNA-binding transcription factor required for hematopoiesis [2],[3] while ETO is a co-repressor molecule expressed in a variety of tissues [4]. In hematopoietic cells, the fusion protein determines a stage specific arrest of maturation and increases cell survival, thus predisposing to leukemia [5].

Full length AML1/ETO is not sufficient to induce AML in mice, and requires treatment with mutagens to induce leukemic transformation [6]–[9]. An alternatively spliced isoform, AML1/ETO9a, isolated from AML patients bearing t(8;21), is instead strongly leukemogenic in mice [10]. *AML1/ETO9a* includes *ETO* exon 9a, which leads to a frameshift of the original coding sequence and consequently to a C-terminally truncated protein. Co-expression of AML1/ETO and AML1/ETO9a results in earlier onset of AML and blocks myeloid cell differentiation at a more immature stage, suggesting the two isoforms could cooperate in patients to induce leukemia.

AML1 and AML1/ETO were originally characterized as DNA binding proteins that recognize, *in vitro*, the conserved core sequence TGT/cGGT [11]. A recent study of AML1 and AML1/ETO binding sites using a 25 bp random oligonucleotide library revealed that AML1/ETO preferentially binds to DNA sequences containing multiple AML1 binding sites [12], suggesting the fusion protein may selectively regulate a subset of AML1 target genes.

AML1/ETO functions as a transcriptional repressor by recruiting NCoR/SMRT/HDAC complexes to DNA through its ETO moiety [4] and blocks AML1-dependent transactivation in various promoter reporter assays, suggesting it may function as a dominant negative regulator of wild-type AML1 [13]–[15]. However, AML1/ETO can also induce the expression of specific target genes: it was initially reported to transactivate *M-CSFR* and *BCL-2* promoters [16],[17] and subsequently, upregulation of other genes was proposed to be crucial for leukemogenesis [18]–[20]. The mechanism for fusion protein-dependent transcriptional activation is not known.

AML1/ETO was recently hypothesized to target DNA through E-box motifs as the result of physical interactions with transcription factors of the E-protein family, in particular HEB/TCF12 [21]. E-proteins (E2A, HEB, and E2.2) are regulators of lymphocytic differentiation, and are involved in acute lymphoblastic leukemias (ALL) [22],[23], while their role in myeloid differentiation and AML has not, to date, been described.

The large-scale determinants of DNA binding by AML1/ETO and the correlation to global transcriptional effects remain to be elucidated. In this study, we present a comprehensive analysis of the DNA binding pattern of AML1/ETO, and its correlation to AML1 and HEB binding sites. The fusion protein preferentially binds to regions that are occupied by the wild-type AML1 transcription factor, without necessarily displacing it. The DNA binding pattern of HEB is reorganized following AML1/ETO expression, and the E-protein re-localizes to AML1/ETO binding regions. Our study provides an accurate description of the genomic distribution of AML1/ETO, and identifies AML1 and HEB as crucial elements for its transcriptional regulatory function.

## Results

### Design of the Study

**Figure 1:**
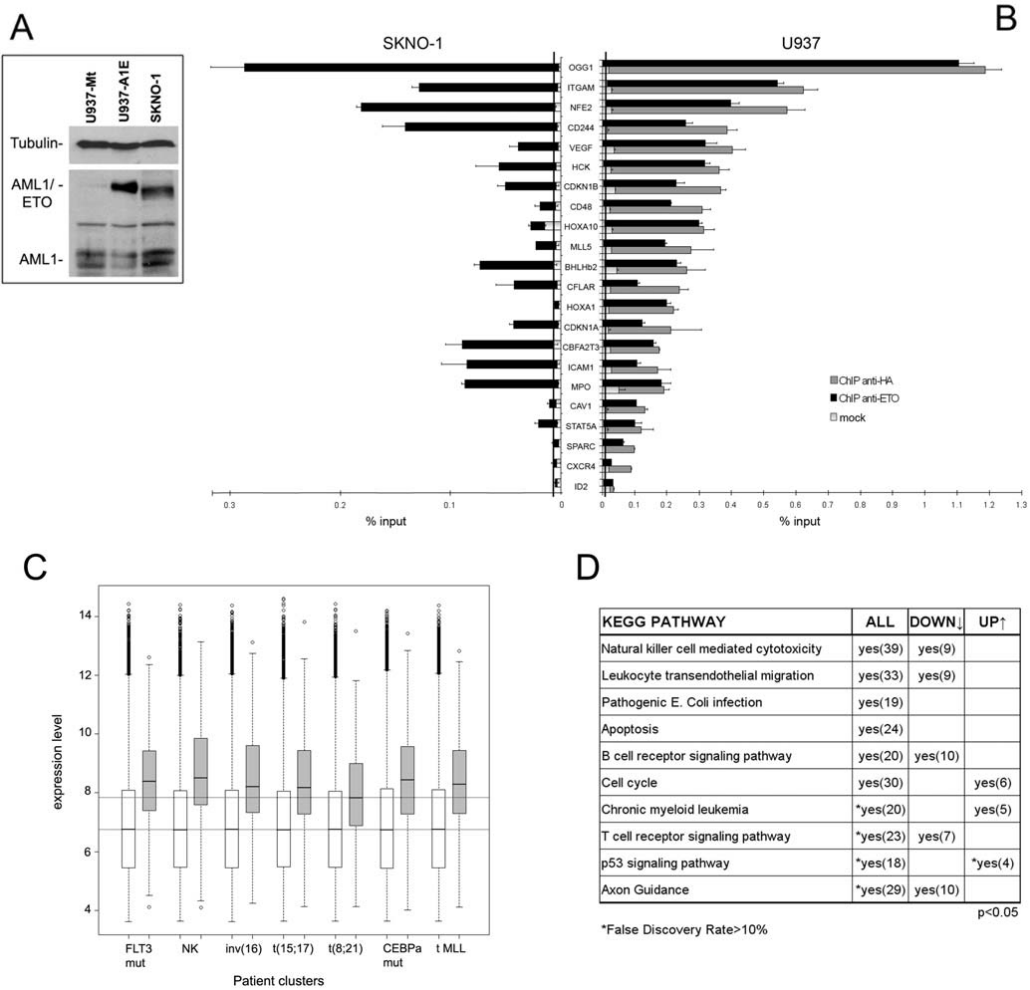
Identification of AML1/ETO binding regions in human promoters. (A) Expression of AML1/ETO protein in U937-AE cells treated for 8 hours with 100 µM ZnSO4 and in patient-derived SKNO-1 cells was investigated by Western blotting using an anti-AML1 antibody. U937-Mt cells were used as negative control. The apparent difference in molecular weight between the two cell lines was due to the HA-tag domain. The two AML1 isoforms are also detected. Anti-tubulin antibody was used for normalization of protein levels. (B) 22 putative target genes were validated by qChIP in U937-AE cells using anti-ETO (black bar) and anti-HA (dark grey bar) antibodies, and in SKNO-1 cells using the anti-ETO antibody. For U937-AE cells, the light grey portion on each bar represents the level of enrichment in the U937-Mt cell line using the anti-HA antibody. In SKNO-1 cells, the mock ChIP was performed using Protein G beads alone. The baseline (represented as a vertical black line on each graph) corresponds to the mean level of enrichment obtained by qChIP on 8 negative control genes (see text and Figure S1). (C) Box-plot representing expression levels in AML samples of AML1/ETO target genes identified by ChIP-chip and expression profiling (approximately 70% of these were included in the array used for the study [28]). Seven representative clusters are shown, including the group of t(8;21) AML. The predominant chromosomal aberration or gene mutation characterizing each cluster is reported below the graph (*FLT3* mut, normal karyotype (NK), inv(16), t(15;17), t(8;21), *CEBPa* mut and t-*MLL* correspond to clusters #2, #5, #9, #12, #13, #15, and #16, respectively, of the original study). White boxes represent expression levels of all genes on the array (lower horizontal line is the median value), grey boxes indicate expression levels of AML1/ETO downregulated targets (higher horizontal line is the median value in t(8;21) AML). (D) Functional pathways enriched in AML1/ETO target genes. The list of all AML1/ETO target genes (Table S1) and the sub-groups of up-regulated and down-regulated genes (Table S4) were functionally annotated using DAVID, and clustered according to the KEGG PATHWAY collection. The table reports categories significantly enriched in the three groups (p-value<0.05, False rate discovery <10%), number of genes retrieved in each pathway are also indicated.

A U937 cell line that conditionally expresses HA-tagged AML1/ETO under the control of the mouse metallothionein (Mt) promoter (U937-AE, [24]) was used for all microarray experiments. U937-Mt cells, which carry the empty vector, served as control. Cells were treated with 100 µM ZnSO4 for 8 hours to induce transgene expression. AML1/ETO protein was expressed at slightly higher levels than those detected in SKNO-1 cells [25], which were derived from an AML patient carrying the 8;21 translocation (Figure 1A). SKNO-1 cells were therefore used to verify the validity of results throughout the study.

Different microarray platforms (Table 1) were exploited to analyze the DNA binding patterns of AML1/ETO, AML1 and the E-protein HEB through chromatin immunoprecipitation (ChIP-chip) and the transcriptional profile of AML1/ETO expressing cells. First, AML1/ETO binding was measured in human promoters and correlated with global transcriptional regulation. Since transcription factors may bind to DNA sequences far from the promoter, AML1/ETO occupancy was also analyzed on a high-resolution tiling array, designed to cover an entire human chromosome. Chromosome 19 (Chr. 19) was chosen due to its relatively small size and high gene density. Although analysis of a single chromosome may introduce a bias, and the distribution of binding sites may be influenced by gene density, these results can be exploited to infer relevant correlations in the binding patterns of specific transcription factors. With this aim, the binding patterns of AML1 and HEB were also analyzed on the Chr. 19 Array. Finally, AML1/ETO-dependent transcriptional regulation of genes localized on Chr. 19 was measured by hybridization with total RNA from U937-AE and U937-Mt cells (expression tiling).

**Table 1:** Microarray experiments included in the study.

Array	Experiment	Cell line(s)	Antibody	Aim
***Genome-wide analyses***				
NimbleGen Human HG17 Promoter Array set	ChIP-chip	U937-AE and U937-Mt	anti-HA	Identification of AML1/ETO binding sites in human promoters
Affymetrix HG-U133 Plus v.2	Gene expression	U937-AE and U937-Mt	—	Identification of genes whose expression is regulated by AML1/ETO
***Analyses on human chromosome 19***				
NimbleGen Chr. 19 Array	ChIP-chip	U937-AE and U937-Mt	anti-HA	Analysis of AML1/ETO binding in different genomic locations
NimbleGen Chr. 19 Array		U937-AE and U937-Mt	anti-ETO	Analysis of AML1/ETO binding in different genomic locations
NimbleGen Chr. 19 Array		U937-AE and U937-Mt	anti-AML1	Correlation of AML1 and AML1/ETO binding patterns
NimbleGen Chr. 19 Array		U937-AE and U937-Mt	anti-HEB	Correlation of HEB and AML1/ETO binding patterns
NimbleGen Chr. 19 Array		U937-AE	anti-H3K4me3	Correlation of AML1/ETO binding pattern with a mark for active transcription
NimbleGen Chr. 19 Array	Gene expression	U937-AE and U937-Mt	—	Correlation of AML1/ETO, AML1 and HEB binding patterns with gene expression

### Identification of AML1/ETO Binding Sites at Human Promoters and Correlation with Transcriptional Regulation

The binding profile of AML1/ETO in human promoters was investigated through ChIP experiments with an anti-HA antibody on lysates of U937-AE and U937-Mt cells. ChIP products were PCR amplified, labeled with Cy3/Cy5 fluorescent dyes and hybridized to the NimbleGen Systems Human HG17 Promoter Array set, which explores 4 kb upstream and 1 kb downstream the transcription start site (TSS) of 24,434 annotated genes. Two biological replicates were prepared and hybridized to independent array sets. A proprietary software (PeakPicker, see Text S1) was used to perform a linear scan across the regions represented on the array, with the aim of identifying clusters of oligonucleotides with positive hybridization signals (from now on defined as “peaks”). Specific AML1/ETO binding regions were selected by first computing all peaks that were present in both experimental replicas, and then discarding those common to the control sample (U937-Mt). 2,726 AML1/ETO peaks were identified in the promoters of 2,513 unique genes, which represent putative targets of the fusion protein (Table S1).

A group of 22 promoters were analyzed by ChIP coupled with qPCR (qChIP, [26]) using the anti-HA or anti-ETO antibodies (ETO protein is not detected in U937 cells). To determine a reliable baseline, qChIP was also performed in U937-AE cells on 8 promoters that did not display AML1/ETO peaks (Figure S1). The anti-HA and anti-ETO antibodies yielded identical data and confirmed the ChIP-chip predictions for 22/22 regions analyzed (Figure 1B). A parallel qChIP experiment was performed on the same promoter regions in SKNO-1 cells: AML1/ETO binding was detected in 17/22 promoters (Figure 1B), suggesting that ChIP-chip data are representative of the genomic distribution of the fusion protein in leukemic blasts.

To assess if the truncated AML1/ETO9a isoform interacts with the same DNA regions as the full length isoform, qChIP was performed on the same 22 promoters using lysates from a U937 cell line that expresses AML1/ETO9a (U937T-AE9a). The binding profile of AML1/ETO9a was similar to that of the long isoform, although relative enrichment levels were not comparable in all cases (Figure S2). This result suggests that the AML1/ETO9a isoform binds to the same DNA regions as full length AML1/ETO, as recently hypothesized based on sequence analysis of binding sites [12].

Affymetrix GeneChip U133 2.0 arrays were used to identify genes differentially expressed in U937-AE cells compared to U937-Mt. Samples were processed and data analyzed as previously described ([24],[27] and Text S1). Expression of 1,316 genes was regulated by AML1/ETO (Table S2): 592 genes (45%) were upregulated and 724 (55%) downregulated. 50 genes were selected for qPCR validation and confirmed microarray results in 90% of cases (Figure S3 and Table S3).

Cross-comparison between ChIP-chip and expression data led to the identification of 358 genes with AML1/ETO peaks in their promoter regions whose transcriptional levels change >1.5-fold (Table S4), of which 247 (69%) downregulated and 111 (31%) upregulated. Expression levels of these genes were then analyzed in a dataset from 285 in vivo AML samples, including 22 patients with t(8;21) [28]. This study had identified 16 groups of AML patients on the basis of gene expression profiles, and the clustering was correlated to the presence of specific chromosomal aberrations and gene mutations [28]. The median expression level of genes downregulated by AML1/ETO was significantly higher (p<0.001) than the median level of other genes on the array (Figure 1C). However, these genes were expressed at a significantly lower level (p<0.05) in the t(8;21) cluster than in other AML clusters (Figure 1C), suggesting they are repressed by the fusion protein in vivo. The same analysis performed on the group of upregulated genes revaled that their median expression level is also higher than that of other genes (data not shown), but a comparative analysis among different AML clusters could not be performed due to the high variability in raw expression values and the small size of the gene list. Taken together, these data suggest that AML1/ETO preferentially binds to and regulates transcription of highly expressed genes.

Functional classification of the 2,513 AML1/ETO target genes according to KEGG molecular interaction networks (http://www.genome.ad.jp/kegg/) [29] highlighted various pathways related to functions of mature leukocytes, such as cytotoxicity, migration and signaling. Interestingly, these were also over-represented in the sub-group of repressed genes, while upregulated targets encoded functions related to cell cycle and chronic myeloid leukemia (Figure 1D and Table S5).

Sequence analysis of AML1/ETO binding regions revealed that the AML1 consensus motif was significantly enriched only in the promoters of downregulated genes (see Text S1 for details on sequence analysis). Conversely, promoters of upregulated genes were characterized by overrepresentation of binding sites for transcription factors not related to myeloid differentiation.

### AML1/ETO Binding Profile on Chromosome 19

AML1/ETO might influence gene expression by binding DNA regions distant from the promoter. We, therefore, analyzed AML1/ETO occupancy across a contiguous genomic region. Two replicas of the Chr.19 Array were hybridized with ChIP products obtained from the U937-AE and U937-Mt cell lines using the anti-HA and anti-ETO antibodies (see Text S1 for data analysis) and 408 AML1/ETO binding peaks were identified (Table S6). The Promoter Array had identified 148 AML1/ETO peaks on chromosome 19; 130 of these were also retrieved with the Chr.19 Array, suggesting that the two platforms yield comparable results.

**Figure 2:**
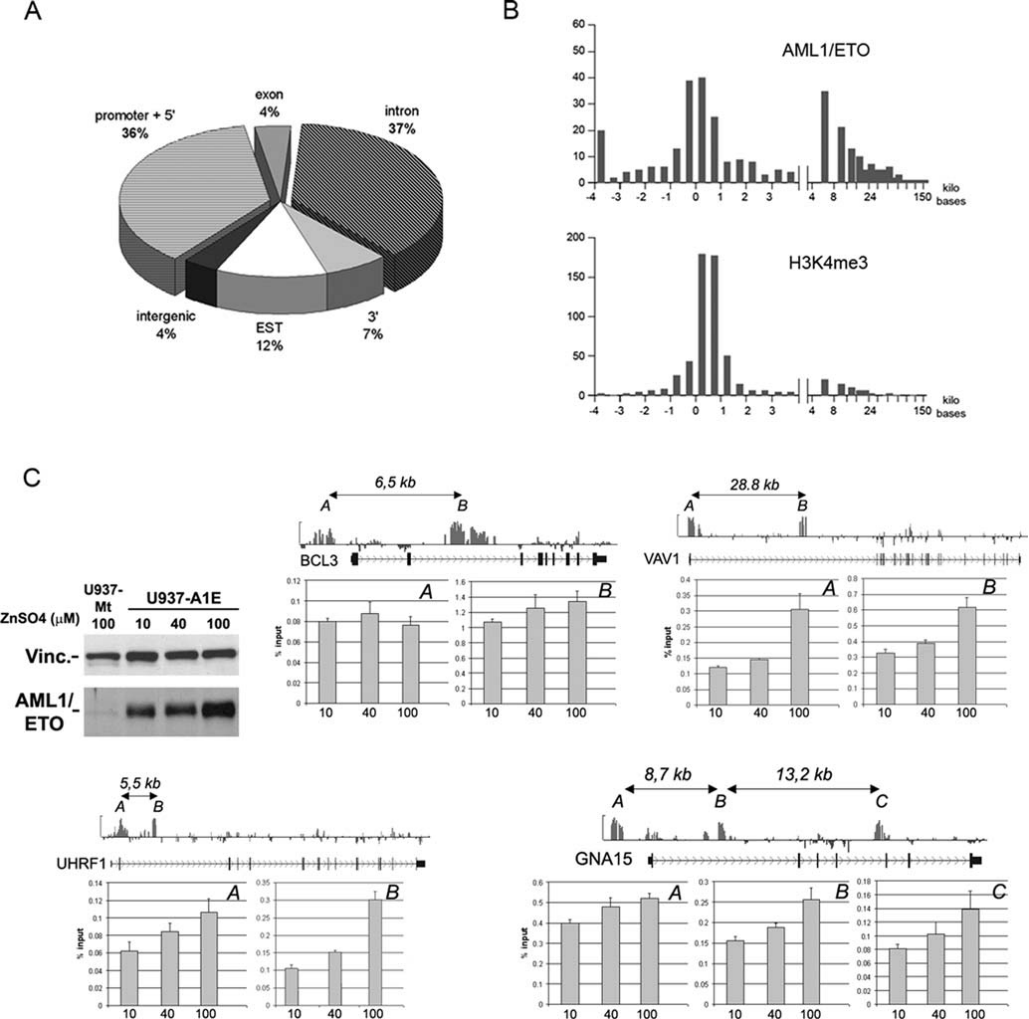
Topography of AML1/ETO binding on chromosome 19. (A) AML1/ETO binding regions on chromosome 19: the pie plot shows the percentage of AML1/ETO peaks in different gene locations. “Promoter+5′” refers to the region comprised between −4 kb and +1 kb respect to the TSS. “3′” refers to the region comprised between the end of the last exon and 4 kb downstream. “Intergenic” refers to all regions that are neither within a gene nor in a region defined as “promoter+5′” or “3′”. Peaks that map in the proximity of transcripts that do not have a GeneBank ID but are present in the EST database were classified as “EST”. “Intergenic” refers to regions without any GeneBank ID or EST. (B) Distribution of AML1/ETO peaks with respect to TSS of annotated genes. Upper panel: distribution of AML1/ETO peaks along the gene body. X-axis indicates the distance from TSS (in kb), Y-axis shows the number of peaks inferred by PeakPicker software. Lower panel: distribution of H3K4me3 peaks, which map mainly within 1 kb from the TSS. (C) Four genes containing AML1/ETO peaks both in promoter and intragenic regions were analyzed for AML1/ETO binding at different fusion protein concentrations. Western blot shows AML1/ETO protein levels in U937-AE cells treated with 10, 40, and 100 µM ZnSO4 for 8 hours. *BCL3*, *VAV1*, and *UHRF1* have two AML1/ETO binding peaks (A = promoter, B = intragenic), whereas *GNA15* has three peaks (A = promoter, B and C = intragenic). For each gene, raw ChIP-chip data aligned to a scheme of the locus are shown above the graphs. AML1/ETO peaks are indicated as A, B, or C, and their relative distances are reported. ChIP experiments were performed using the anti-HA antibody on U937-AE treated with ZnSO4 at the doses indicated below the graphs. Variations in relative enrichment do not depend on location of peaks.

343/408 (84%) AML1/ETO peaks mapped to 254 known genes, and can be further sub-localized as follows: 36% are within promoters (defined as −4 kb to +1 kb from the TSS), 4% within exons, 37% inside introns and 7% in 3′ sequences in proximity of the gene (Figure 2A). Of the 254 AML1/ETO target genes, 45% have binding sites only in the 5′ regulatory region, 46% have binding sites only within the gene body (exons, introns or 3′ region), and 9% were bound both in the promoter and in the gene body.

The distribution of AML1/ETO peaks in U937-AE cells was compared with that of histone mark H3K4me3, which consistently associates with active promoters. ChIP products obtained with an anti-H3K4me3 antibody on lysates of U937-AE cells were hybridized to the Chr. 19 Array. H3K4me3 peaks clustered in the proximity of TSS (Table S7), while AML1/ETO peaks clustered both near TSS and in distant locations (>5 kilobases downstream, Figure 2B), confirming that the fusion protein does not bind exclusively to promoters.

AML1/ETO binding outside of promoter regions may depend on protein concentration. Increasing doses of ZnSO4 (10, 40 and 100 µM) were, therefore, used to titrate fusion protein levels, and qChIP was performed on both promoter and downstream binding sites in four of genes that present peaks in both locations (Figure 2C). AML1/ETO binding was not homogeneous in the different gene locations: enrichment levels were higher in the intragenic peaks of three genes (*BCL3*, *VAV1* and *UHRF1*) and lower in one gene (*GNA15*) (Figure 2C); however, AML1/ETO protein levels did not affect the binding pattern.

### AML1 Binding Profile on Chromosome 19

**Figure 3:**
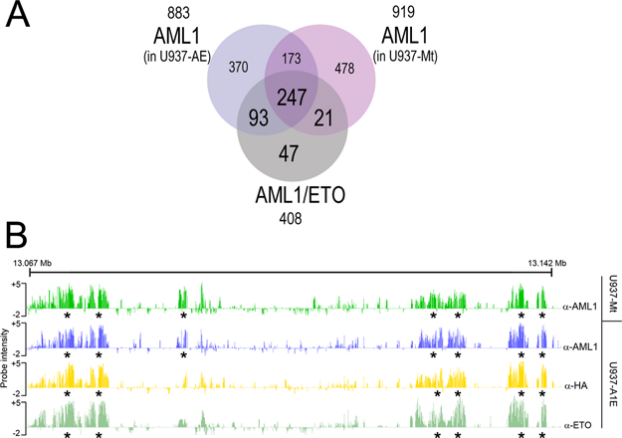
AML1 binding pattern on chromosome 19. (A) Venn diagram representing the overlap of AML1 binding sites in U937-AE and U937-Mt cells with AML1/ETO binding sites on chromosome 19. Physical overlaps >20% were considered significant. (B) Representative screenshot of AML1 and AML1/ETO occupancy on chromosome 19. A window of 80 kb is shown. The two top lanes represent the AML1 binding profile in the control cell line U937-Mt and in U937-AE, respectively. The two bottom lanes represent AML1/ETO binding patterns obtained with anti-HA and anti-ETO antibodies. Asterisks indicate peaks identified by PeakPicker software.

We next investigated if the binding profile of AML1/ETO reflects that of the native AML1 transcription factor. A ChIP-chip experiment was performed on the same cell lines using an anti-AML1 antibody that does not cross-react with AML1/ETO, since it recognizes the AML1 C-terminal portion, which is lost in the fusion protein. ChIP products were hybridized to Chr.19 Arrays: 883 AML1 peaks were identified in U937-AE cells and 919 peaks in U937-Mt cells (Table S8). 420 of the AML1 peaks were common to both cell lines, demonstrating that 46% of AML1 binding sites remain occupied by AML1 after expression of the fusion protein (Figure 3A).

The extent of overlap between AML1/ETO and AML1 binding regions was quantified by comparing peak coordinates (Table S6 and Figure 3A–3B). Of the 408 AML1/ETO peaks identified on Chr. 19, 83% (340/408) intercept AML1 peaks in U937-AE cells (Figure 3A). Only 5% (21/408) of AML1/ETO binding occurs in regions where AML1 was localized in U937-Mt cells but not in U937-AE cells. These data suggest that AML1/ETO preferentially binds to regions occupied by AML1 and does not function primarily by displacing AML1 from its binding sites *in vivo*.

### HEB Binding Profile on Chromosome 19

**Figure 4:**
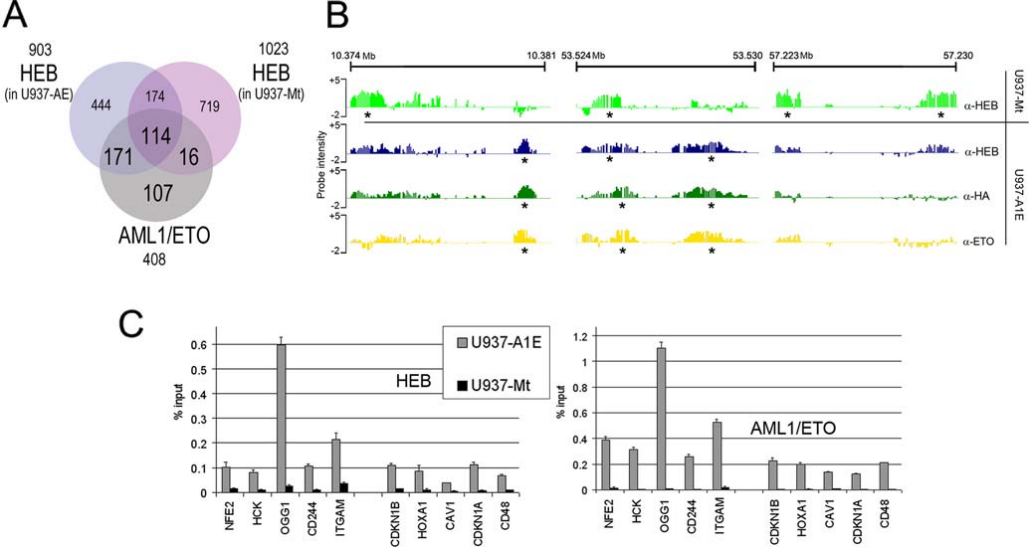
HEB binding pattern on chromosome 19. (A) Venn diagram representing the overlap of HEB binding sites in U937-AE and U937-Mt cells with AML1/ETO binding sites on chromosome 19. Regions with at least 20% physical overlap were considered significant. (B) Screenshots of HEB and AML1/ETO occupancy on chromosome 19. Three representative regions show the displacement of HEB upon expression of AML1/ETO and highlight the similarity of the binding patterns of the two proteins in U937-AE cells. The two top lanes represent HEB binding profile in U937-Mt and in U937-AE. The two bottom lanes represent AML1/ETO binding patterns obtained with anti-HA and anti-ETO antibodies. Asterisks indicate peaks identified by PeakPicker software. (C) qChIP with an anti-HEB antibody analyzing the promoter of 10 genes regulated by AML1/ETO (5 downregulated: *NFE2*, *HCK*, *OGG1*, *CD244*, and *ITGAM*; and 5 upregulated: *CDKN1B*, *HOXA1*, *CAV1*, *CDKN1A*, and *CD48*) shows increased amounts of HEB in U937-AE (left graph). Right graph shows ChIP analysis of AML1/ETO protein on the same regions.

Detailed sequence analysis using both supervised and unsupervised methods revealed a specific sequence signature associated with genomic occupancy of AML1/ETO, which includes a significant enrichment for AML1 and HEB binding sites (Text S1). The finding that AML1/ETO binding regions often contain the consensus motif for HEB is of particular interest, since HEB associates with AML1/ETO *in vivo* [21]. The interaction of endogenous HEB with AML1/ETO in U937-AE cells was confirmed through co-immunoprecipitation experiments (Figure S4) and the DNA binding pattern of HEB was investigated on the Chr. 19 Array through ChIP-chip analysis using an anti-HEB antibody. 903 HEB peaks were identified in U937-AE cells and 1023 peaks in U937-Mt cells (Table S9), and there was a massive redistribution of HEB binding regions in cells expressing AML1/ETO (Figure 4A–4B): in fact, only 288/1023 (28%) peaks retrieved in U937-Mt cells are also identified U937-AE, and 615/903 (67%) of HEB peaks retrieved in U937-AE represent novel binding sites.

Many HEB peaks in U937-AE cells coincide with AML1/ETO binding regions: of the 408 AML1/ETO peaks, 285 (70%) are also recognized by HEB (Table S6 and Figure 4A). To validate this finding, qChIP was performed on 5 downregulated and 5 upregulated genes identified as AML1/ETO targets on the Promoter Array. A significant increase of promoter-bound HEB was detected, and enrichment levels were proportional to those of AML1/ETO (Figure 4C). The correlation between AML1/ETO and HEB binding was also investigated in SKNO-1 cells by qChIP on 22 promoters (Figure S5). Enrichment of HEB was detected in the same promoters bound by AML1/ETO (Figure 1B), confirming a significant correlation in the binding pattern of the two transcription factors in AML cells expressing the fusion protein.

In the absence of AML1/ETO, HEB and AML1 localize to the same genomic regions in approximately 25% of their global binding sites, suggesting they may be involved in co-regulation of common target genes (Text S1 and Figure S6). Interestingly, AML1/ETO expression results in displacement of HEB and AML1 from these common regions at lower frequency than in genomic locations where these transcription factors do not co-localize (Figure S6).

### Correlation between AML1, HEB, and AML1/ETO Binding Profiles and Gene Expression

The correlation between gene expression and DNA binding pattern of AML1/ETO, AML1 and HEB was investigated by expression tiling. The Chr. 19 array was hybridized with RNA derived from U937-Mt and U937-AE cells and expression levels of the 1305 genes on chromosome 19 were calculated as described in Text S1 (Table S10). The median expression value of the 254 genes associated with AML1/ETO peaks resulted significantly higher than that of the remaining 1051 genes on chromosome 19 (Figure S7), confirming our previous observation that AML1/ETO preferentially binds to genomic regions containing actively transcribed genes (Figure 1C). Comparison of expression levels in U937-AE and U937-Mt cells brought to the identification of 52 regulated genes (fold change >1.5, p-value<0.05). AML1/ETO binds to genomic regions in the proximity of 24 of these (23 repressed, 1 induced). All of them were also associated with AML1 peaks and 23/24 with HEB peaks (Table S10). The remaining 28 regulated genes did not display AML1/ETO binding. However, 10 of these contain HEB binding regions in wild-type conditions, and HEB is displaced in the presence of AML1/ETO (Figure S8). Therefore, the expression level of HEB target genes can be modified by AML1/ETO expression, suggesting that transcriptional regulation determined by AML1/ETO may partly derive from displacement of HEB from its native binding sites.

### HEB Protein Levels Increase in AML1/ETO-Expressing Cells

**Figure 5:**
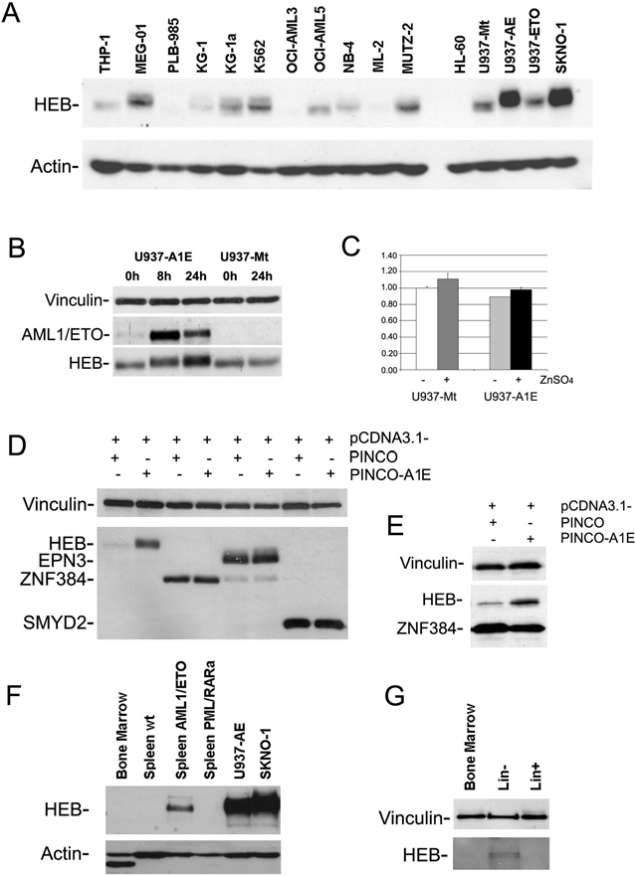
HEB protein levels increase in AML1/ETO-expressing cells. (A) Expression of HEB protein in a panel of human myeloid cell lines: 30 µg of total cell lysates were analyzed by Western blot with an anti-HEB antibody. AML1/ETO positive cells (U937-AE and SKNO-1) express higher levels of HEB. U937 cell lines were induced with ZnSO4 for 24 hours. An anti-actin antibody was used for normalization of protein levels. (B) U937-AE cells were treated for 8 and 24 hours with 100 µM ZnSO4. AML1/ETO protein levels peak after 8 hours. HEB protein levels increase after AML1/ETO expression. No modification in HEB levels is detectable after ZnSO4induction of control U937-Mt cells. (C) *HEB* mRNA levels in U937-AE and U937-Mt cells prior to and after 8 hours of 100 µM ZnSO4 treatment are shown (*GAPDH* normalized). AML1/ETO does not modify the levels of *HEB* transcript. (D) HEB protein levels in HeLa cells increase when pCDNA3.1-FLAG-HEB is co-transfected with PINCO-AML1/ETO expression vector. This effect is not seen after co-transfection of AML1/ETO with other genes cloned into the same vector (pCDNA3.1-FLAG). All proteins were revealed by anti-FLAG immunoblot. (E) Co-transfection of PINCO-AML1/ETO with both pCDNA3.1-FLAG-HEB and pCDNA3.1-FLAG-ZNF384 results in a specific increase of HEB protein levels. (F) Expression of HEB protein in mouse tissues. HEB is not detected in wild-type murine bone marrow or spleen. AML blasts derived from the spleens of AML1/ETO transgenic mice express HEB, whereas blasts from PML/RARα transgenics do not. (G) HEB protein levels were analyzed in total bone marrow, Lin− and Lin+ cellular compartments form wild-type mice. Detectable levels of HEB are found only in the Lin− fraction of murine bone marrow.

Our data suggest HEB may play a role in AML1/ETO-dependent transcriptional regulation. However, not much is known concerning HEB protein expression in the myeloid lineage. The level of HEB protein in a series of leukemic cell lines of myeloid origin, including AML1/ETO expressing cells, was therefore investigated (Figure 5A). Notably, HEB is highly expressed in AML1/ETO positive cell lines (SKNO-1 cells and ZnSO4-induced U937-AE, Figure 5). Other cell lines, including U937-Mt and a U937 clone expressing the ETO moiety of the fusion protein (U937-ETO), display significantly less HEB protein (Figure 5A). To exclude cell line specific effects, expression levels of AML1/ETO and HEB were analyzed in U937-AE and U937 Mt cells before and after induction with 100 µM ZnSO4 and (Figure 5B). HEB protein levels were similar in the two uninduced cell lines, and increased after ZnSO4 treatment only in U937-AE cells. *HEB* mRNA levels were, instead, identical in U937-AE and U937-Mt cells prior to and after ZnSO4 induction (Figure 5C), suggesting that the increase in HEB protein is due to post-translational regulatory mechanisms.

The effect of AML1/ETO expression on HEB protein levels was confirmed in a different cellular context. Co-transfection of HeLa cells with PINCO-AML1/ETO and pCDNA3.1-FLAG-HEB expression vectors showed that HEB protein levels were significantly higher than in control cells transfected with pCDNA3.1-FLAG-HEB and PINCO (Figure 5D). This effect is not mediated by induction of pCDNA3.1-driven transcription, since expression levels of other genes inserted into the same vector were not affected by co-expression with PINCO-AML1/ETO (Figure 5D). To exclude artifacts due to differences in transfection efficiency, PINCO-AML1/ETO was co-transfected with both pCDNA3.1-FLAG-HEB and pCDNA3.1-ZNF384 (Figure 5E). Only HEB protein levels were higher in cells expressing AML1/ETO, while ZNF384 levels were not significantly affected by the fusion protein.

The correlation between AML1/ETO and HEB protein levels was next analyzed in primary cells from AML mouse models. HEB protein is not detected in wild-type bone marrow cells, or in normal splenocytes (Figure 5F). Leukemic blasts from AML1/ETO mice expressed significant levels of HEB (Figure 5F), as opposed to blasts from PML/RARα transgenic mice, which provide another model of AML. These data enforce the idea that HEB plays a role selectively in t(8;21) leukemia.

Although total bone marrow cells did not express HEB (Figure 5F), the more undifferentiated fraction (Lin− cells) had a detectable, albeit low, amount of HEB protein, as opposed to the more abundant Lin+ differentiated cells (Figure 5G). This result suggests that HEB might be present only in a subset of early progenitors.

## Discussion

The analysis of transcription factor binding patterns using genome-wide approaches can serve not only to identify direct target genes, but also to discover interactions among transcription factors that could help in defining disease-linked regulatory networks. We investigated the genome-wide binding profile of AML1/ETO at human promoters and in a contiguous genomic region, and its correlation with AML1 and HEB, with the aim of understanding the determinants of its transcriptional regulatory function.

In U937 cells, AML1/ETO binds to the promoter regions of 2,513 non-redundant genes, and causes transcriptional changes in 358 of them. Of these, 70% are repressed, including many genes involved in neutrophilic/myeloid differentiation (i.e. *MPO*, *HCK*, *FYN*, *GADD45B*, *ITGAM*, *ITGB2*). On the other hand, 30% of target genes are induced, including *HOX* genes (*HOXA10*, *HOXA5*, *HOXC6*) and the cell cycle regulators *CDKN1A* and *CDKN1B*. *CDKN1A* is of particular interest since it is know to regulate maintenance of quiescent hematopoietic stem cells [30] and also modulates leukemic development in mouse models of t(8;21) ([31], PG Pelicci, subitted for publication). The AML1 consensus sequence is only enriched in downregulated genes, whereas upregulated genes show enrichment in binding sites for other transcription factors. Therefore, a proportion of AML1/ETO binding to DNA may be achieved independently from the AML1 motif recognition.

AML1/ETO has been hypothesized to function as dominant negative of its wild type component AML1 [13]–[15]. Analysis of the DNA binding pattern of AML1 showed that the two transcription factors often bind to the same DNA regions. In many cases AML1/ETO target genes represent native AML1 targets, but the presence of the fusion protein does not displace the wild-type protein. In other cases, the two proteins are present in genomic regions where AML1 is normally not found. Therefore, although the two proteins do not physically associate (data not shown), AML1/ETO expression is capable of driving AML1 to new sites on the genome, perhaps by rendering them accessible. The functional consequences of the co-localization of AML1 and AML1/ETO on transcriptional regulation remain to be elucidated.

The E-protein HEB has been described as an interactor of both AML1/ETO and ETO [21]. It was hypothesized that through this interaction, AML1/ETO could be redirected to E-protein target genes, possibly deregulating their expression. We found that the binding pattern of HEB in U937 cells is disrupted upon expression of AML1/ETO, and the fusion protein recruits HEB to its binding regions. Co-occurrence of HEB and AML1/ETO in promoters was also detected in the SKNO-1 cell line, demonstrating that this phenomenon is not peculiar to the cellular system under analysis. The specific repositioning of HEB to AML1/ETO binding sites is also supported by sequence analysis, which identifies the AML1 motif in HEB binding regions only in cells expressing AML1/ETO.

More information concerning the complexity of AML1/ETO binding can be obtained by looking simultaneously at the binding patterns of AML1 and HEB in the presence and absence of AML1/ETO. The first observation is that these two transcription factors share a proportion of target genes in wild-type conditions, pointing to common regulatory functions in hematopoiesis. When they bind to common target sites, AML1 and HEB are less prone to be displaced by AML1/ETO expression, and these regions represent preferential binding sites for the fusion protein. Furthermore, regulation of gene expression by AML1/ETO is significantly associated with the co-occurrence of AML1 and HEB, suggesting that common binding regions for the three transcription factors are of relevance to AML1/ETO function.

A proportion of genes regulated by AML1/ETO did not display binding of the fusion protein in proximity of the gene. Our results demonstrate that some of these genes contain regions bound by HEB in wild-type conditions, and that this binding was no longer present in cells expressing AML1/ETO. Therefore, indirect transcriptional regulation determined by AML1/ETO may be partially due to displacement of HEB from its native target genes, and interference with HEB-dependent gene expression.

In the hematopoietic system, HEB has been studied in lymphoid differentiation: loss of function experiments have shown that HEB is relevant in the early stages of B-cell and T-cell development [32]. We show that HEB is expressed in the Lin− compartment of murine bone marrow, and is absent in more differentiated myeloid cells. HEB expression appears to be necessary for survival of cells expressing the fusion protein (A. Gardini and M. Alcalay, unpublished), suggesting that the translocation may only be viable in cells that express HEB, such as early progenitors or stem cells. Furthermore, HEB protein levels increase in the presence of AML1/ETO, whereas *HEB* mRNA levels remain unchanged, implying that expression of the fusion protein is associated with stabilization of HEB through unknown post-translational mechanisms. Therefore, it is possible that the HEB-expressing subpopulation of progenitor/stem cells might contain the target cell for AML1/ETO, which would then expand the compartment of HEB-expressing cells and sustain HEB protein levels by modulating post-translational regulatory mechanisms.

Our study represents the first comprehensive analysis of AML1/ETO global genomic occupancy and provides a correlation to its effect on gene expression. Our results point to a pivotal role for AML1 and HEB in t(8;21) myeloid leukemogenesis.

## Materials and Methods

### Cell Lines

The U937 AML1/ETO-HA#9 clone (U937-AE) was generated by stable transfection of AML1/ETO-HA cDNA cloned in the inducible pSG-MtNEO plasmid vector as already described [24]. The U937 ETO#15 clone (U937-ETO) carries the cDNA portion of ETO retained in the fusion gene in pSG-MtNEO (M. Alcalay and P.G. Pelicci, unpublished). A bulk population of U937 cells transfected with the empty pSG-MtNEO vector (U937-Mt) was used as control. Cell lines were treated for 8 hours with 100 µM ZnSO4 to allow expression of the transgene.

U937-AML1/ETO9a cells (U937T-AE9a) were generated by stable transfection of U937T cells [33] with HA-tagged AML1/ETO9a cloned in the inducible pUHD10.3 tet-off vector. Cells were grown in the presence of 1 µg/ml tetracyclin. HA-AE9a induction was reached after 24 h of tetracyclin withdrawal.

U937 cells were grown in RPMI-1640 supplemented with 10% FCS and 2 mM glutamine at 37°C in a humidified atmosphere containing 5% CO2. HeLa cells were grown in DMEM medium supplemented with 10% FCS and 2 mM glutamine. Myeloid cell lines used for Western Blotting were grown according to standard procedures.

### Antibodies

Immunopurified anti-HA from clone 12CA5 was used against AML1/ETO. Commercially available antibodies against ETO (Santa Cruz sc-9737), AML1/RUNX1 (Abcam ab23980), HEB (Santa Cruz sc-357), H3K4me3 (Abcam ab8580) were used in ChIP assay and Western blot. The anti-AML1/RHD (Oncogene PG285) antibody, recognizing both wild-type AML1 and AML1/ETO, anti-alpha-tubulin (SIGMA T9026), anti-vinculin (SIGMA V9131) and anti-actin (SIGMA A4700) were used for Western blotting.

### Chromatin Immunoprecipitation

Cells were cross-linked with 1% formaldehyde for 10 minutes at room temperature, harvested and washed twice with 1× PBS. Pellet was resuspended in ChIP lysis buffer (150 mM NaCl, 1% Triton-X 100, 0,1% SDS, 500 µM DTT, 10 mM Tris-HCl, 5 mM EDTA) and sonicated to obtain an average chromatin length of 500 bp. 5×10^6^ cells were used for each IP and incubated at 4°C overnight with the antibody of interest previously coated on Dynabeads Protein A magnetic beads (Invitrogen, USA). For anti-ETO ChIP, Protein G Sepharose beads were used (Zymed, USA).

Beads were then washed twice with each of the following buffers: Mixed Micelle Buffer (150 mM NaCl, 1% Triton-X 100, 0,2% SDS, 20 mM Tris-HCl, 5 mM EDTA, 65% sucrose), LiCl/detergent wash (250 mM LiCl, 0.5% Na deoxycholate, 0,5% NP-40, 10 mM Tris-HCl, 1 mM EDTA), Buffer 500 (500 mM NaCl, % Triton-X 100, 0.1% Na deoxycholate, 25 mM HEPES, 10 mM Tris-HCl, 1 mM EDTA) an a final wash was performed with 1× TE. Finally, beads were resuspended in 1× TE containing 1% SDS and incubated at 65°C for 10 minutes to elute immunocomplexes. Elution was repeated twice, and the samples were further incubated overnight at 65°C to reverse cross-linking, along with the untreated input (2,5% of the starting material). After treatment with 0,5 mg/ml proteinase K for 3 hours, DNA was purified with Wizard SV Gel and PCR Clean-up system (Promega, USA) according to manufacturer's protocol and eluted in nuclease-free water. ChIP products and input DNA were used for quantitative PCR or further treated for ChIP-chip.

### ChIP-chip

The NimbleGen Systems Human HG17 Promoter Array set from NimbleGen catalogue, which contains >700,000 probes exploring 4 kb upstream and 1 kb downstream of 24,434 annotated genes, were used for genome-wide identification of AML1/ETO binding sites. In addition, custom designed high-density oligonucleotide arrays, containing ∼360,000 isothermal probes (from 50 to 60 bp long) contiguously tiled along chromosome 19, were produced by NimbleGen Systems (Madison, Wisconsin, USA). Non-repetitive sequences of chromosome 19 available from UCSC Hg.17 were used for the probe design.

ChIP products were obtained as described above. 80% of a single ChIP product and 40 ng of the input were further treated for array hybridization as described by Kim et al. [34]. Briefly, samples were blunt-ended then ligated to the annealed linker oligonucleotides JW102: GCGGTGACCCGGGAGATCTGAATTC and JW103: GAATTCAGATC. DNA was amplified in a 1^st^ LM-PCR reaction using oligo JW102 and Phusion DNA Polymerase (FynnZyme, Oy, Finland), and PCR products were purified with Wizard SV Gel and PCR Clean-up system. 200 ng were used as template in a 2^nd^ LM-PCR. 4 µg of purified DNA were labelled by NimbleGen Services using random priming and Cy3/Cy5 fluorescent dyes. Input DNA and ChIP DNA were labeled with different fluorophores and co-hybridized on the same array. For every probe, results were rated as log2 (ratio ChIP/input). Labeling and hybridization were performed by NimbleGen Services, Reykjavik, Iceland. Data were visualized and images extracted using SginalMap software (NimbleGen Systems).

### ChIP-chip Data Analysis

A perl script called PeakPicker (M. Cesaroni et al, manuscript submitted) was developed as a tool to perform peak analysis on different types of ChIP-chip platforms. Briefly, PeakPicker identifies binding regions (peaks) by centering a sliding window of user-defined size around every probe of the array, then picking out the number of probes within the window that are above a given percentile (see Text S1). For the analysis of Promoter Array data, since probes are more sparse (110 bp interval), a lower stringency analysis was used: 90^th^ percentile, window of 1000 bp, |log(p-value)|>2. For the analysis of Chr.19 Array data, stringent parameters were applied: 98^th^ percentile, window of 500 bp, |log(p-value)|>7.

PeakPicker generates a tab file representing a list of binding peaks sorted by their first and last nucleotide mapped on UCSC Hg.17. Comparison between two lists of peaks is obtained by measuring the percentage of physical overlap. Peaks with at least 20% physical overlap are considered co-occurrent. To generate the high stringency list of 408 AML1/ETO binding peaks on Chr.19, we compared anti-ETO and anti-HA lists using a threshold of 60% peak overlap.

### qPCR Analysis of ChIP Samples

All reactions were performed with the following reagents: 0.4 µM of each primer, 12.5 µl of SYBR Green PCR Master MIX (Applera, USA), and a fixed volume of template DNA in a final volume of 25 µl. Thermal cycling parameters were: 2 minutes at 50°C, followed by 10 minutes at 95°C, followed by 40 cycles of 15 seconds at 95°C, and 1 minute at 60°C. We used 1/40 of the eluted DNA from both ChIP samples and input. Oligonucleotides were designed to validate regions occupied by AML1/ETO according to bioinformatics analysis of NimbleGen Promoter Array (see Text S1 for oligo sequences). The amount of immunoprecipitated DNA relative to that present in total input chromatin was calculated as described by [26].

### RNA Extraction, Affymetrix GeneChip Hybridization, and Analysis

Total RNA was extracted using the RNeasy Mini Kit (QIAGEN, Valencia, California, USA). For each of the U937 cell lines (AE and Mt), three independent RNA extractions were performed, and an equal quantity of each of the three RNA preparations was then mixed to generate an RNA pool for each sample.

RNA pools were labeled and hybrydized to the Affymetrix HG-U133 Plus 2.0 array (Affymetrix, USA). Results derived from U937-AE cells (sample) were compared to results from U937-Mt (baseline) cells by “comparative analysis” with GCOS software. Data were then analyzed using GenePicker software [35] as previously described [24], using a fold change cutoff >1.5 and a p-value of 0.05. For details on Affymetrix data generation and processing see Text S1.

50 regulated genes were chosen for validation by qRT-PCR on an independent set of RNAs. qPCR was performed as already described for ChIP samples, using 15 ng of cDNA reverse transcribed with Super Script III (Invitrogen, USA) according to manufacturer's protocol (see Table S3 for primer sequences).

### Functional Classification of AML1/ETO Target Genes

Functional classification was performed starting from the list of AML1/ETO target genes (Table S1) and the sub-group of regulated genes (Table S4). Gene lists were functionally annotated using DAVID (http://david.abcc.ncifcrf.gov) [36], and clustered according to the KEGG PATHWAY collection [29].

### Co-Immunoprecipitation

U937-AE and U937-Mt were treated with 100 µM ZnSO4, collected and resuspended in CoIP buffer (20 mM Tris HCl pH 7.5, 300 mM NaCl, 1% Triton, 20% Glycerol, 1 mM EDTA, 1 mM EGTA, 2.5 mM Na Pyrophosphate, 0.5 mM DTT). After brief sonication, lysates were cleared by centrifugation at 13,000 rpm and incubated with the indicated antibody and Protein A sepharose beads (Bio-Rad, Hercules, California, USA). Immunoprecipitates were washed 4 times in the CoIP buffer, eluted and separated by electrophoresis on denaturing SDS-PAGE. Immunoblotting was performed according to standard procedures.

### Mouse Cell Lysates

Bone marrow cells were harvested from 8- to 10-week-old 129SvEv mice and treated for purification of undifferentiated cells (Lin−). After centrifugation through a Ficoll gradient, mononucleated cells were enriched for progenitors by affinity depletion of cells presenting myeloid, erythroid, and lymphoid differentiation markers using commercially available reagents (Stem Cell Technologies, Vancouver, BC, Canada). Depleted cell were also collected as the differentiated subpopulation (Lin+).

Leukemic mice were generated by retroviral transduction of PINCO-AML1-ETO or PINCO-PML/RAR into Lin− cells, as described [37]. Briefly, Lin− cells were infected in 24-multiwell plates coated with retronectin. GFP-positive cells were FACS-sorted (FACSVantage instrument, BD) and reinoculated into lethally irradiated (10 Gy) syngeneic mice (2×10^5^ Lin−/mouse). After 4 weeks, animals transduced with AML1/ETO expressing cells were treated with N-ethyl-N-nitrosourea (50 mg/kg), as described [38]. AML blasts cells were harvested from the spleens of leukemic animals (with >80% infiltration of leukemic cells) by centrifugation through Ficoll gradient, and lysed for Western blot analysis.

### HEB Cloning and Transfection Assays

The full-length HEB isoform (RefSeq accession number: NM_207036) was isolated from U937 cells by PCR amplification of cDNA, using the following primers: f-ATGGATCCTAATCCCCAGCAACAACGC (contains an additional BamHI protruding 5′ tail) and r- ATCTCGAGTTACATATGACCCATAGG (contains an additional Xho protruding 5′ tail). PCR reactions were run on an agarose gel, extracted, purified and inserted into BamHI-Xho digested pCDNA3.1 (Invitrogen), which contains an in-frame FLAG tag sequence at the 5′.

A FLAG-pCDNA3.1 vector containing full length ZNF384 was also generated by PCR-cloning using f-ATGGTACCAGAAGAATCTCACTTC and r-ATGAATTCCTAAGAGCTGGCCAGG primers. FLAG-pCDNA3.1 vectors containing SMYD2 and EPN3 were a gift from Bruno Amati and Pier Paolo Di Fiore, respectively. PINCO and PINCO-AML1/ETO vectors used in the co-transfection studies were already published [39].

Transfection in HeLa cells was performed using Lipofectamine (Invitrogen) and 500 ng of each plasmid (1 well in a 6-well plate), diluted in serum-free DMEM culture medium. Cells were collected and lysed for Western Blotting 24 hours after transfection.

### Accession Numbers

Microarray data included in this manuscript have been deposited in GEO, (http://www.ncbi.nlm.nih.gov/geo/), and can be retrieved as Data Series with accession number GSE10537.

## Supporting Information

Table S1 AML1/ETO target regions in human promoters identified by ChIP-chip on the NimbleGen Human Promoter Array set.
(0.32 MB XLS)

Table S2 Genes regulated by AML1/ETO in U937-AE cells.
(0.23 MB XLS)

Table S3 Validation of genes regulated by AML1/ETO.
(0.03 MB XLS)

Table S4 Genes that present binding of AML1/ETO and whose expression is regulated in U937 cells.
(0.08 MB XLS)

Table S5 Functional classification of AML1/ETO target genes.
(0.13 MB XLS)

Table S6 408 regions on chromosome 19 bound by AML1/ETO identified on the Chr. 19 Array.
(0.12 MB XLS)

Table S7 H3K4me3 regions on chromosome 19 in U937-AE cells.
(0.15 MB XLS)

Table S8 Binding regions of the endogenous AML1 transcription factor on chromosome 19 in U937-AE cells and U937-Mt cells.
(0.36 MB XLS)

Table S9 Binding regions of the endogenous HEB transcription factor on chromosome 19 in U937-AE and U937-Mt cells.
(0.41 MB XLS)

Table S10 Expression levels of genes on chromosome 19 in U937-Mt and U937-AE cells.
(0.36 MB XLS)

Figure S1 Calculation of baseline values for qChIP experiments.
(0.10 MB DOC)

Figure S2 qChIP analysis of AML1/ETO9a binding on AML1/ETO target regions.
(0.18 MB DOC)

Figure S3 Validation of transcriptional regulation of AML1/ETO target genes identified by gene expression profiling.
(0.17 MB DOC)

Figure S4 AML1/ETO interacts with the E-protein HEB in U937-AE cells.
(0.10 MB DOC)

Figure S5 qChIP analysis of HEB binding on AML1/ETO target regions in SKNO-1 cells.
(0.12 MB DOC)

Figure S6 Rearrangement of HEB and AML1 binding patterns in AML1/ETO expressing cells.
(0.88 MB DOC)

Figure S7 AML1/ETO preferentially binds in the proximity of expressed genes.
(0.10 MB DOC)

Figure S8 Displacement of HEB from its native binding sites is associated to transcriptional regulation.
(0.20 MB DOC)

Text S1 Supplementary data and methods.
(1.58 MB DOC)
