# Supplementary material for: AML1/ETO Oncoprotein Is Directed to AML1 Binding Regions and Co-Localizes with AML1 and HEB on Its Targets
Source: PLoS Genet. 2008 Nov 28;4(11):e1000275. doi: 10.1371/journal.pgen.1000275 (PMC2577924; doi:10.1371/journal.pgen.1000275)
Supplement: Figure S1 — Calculation of baseline values for qChIP experiments. (0.10 MB DOC) [file pgen.1000275.s011.doc]

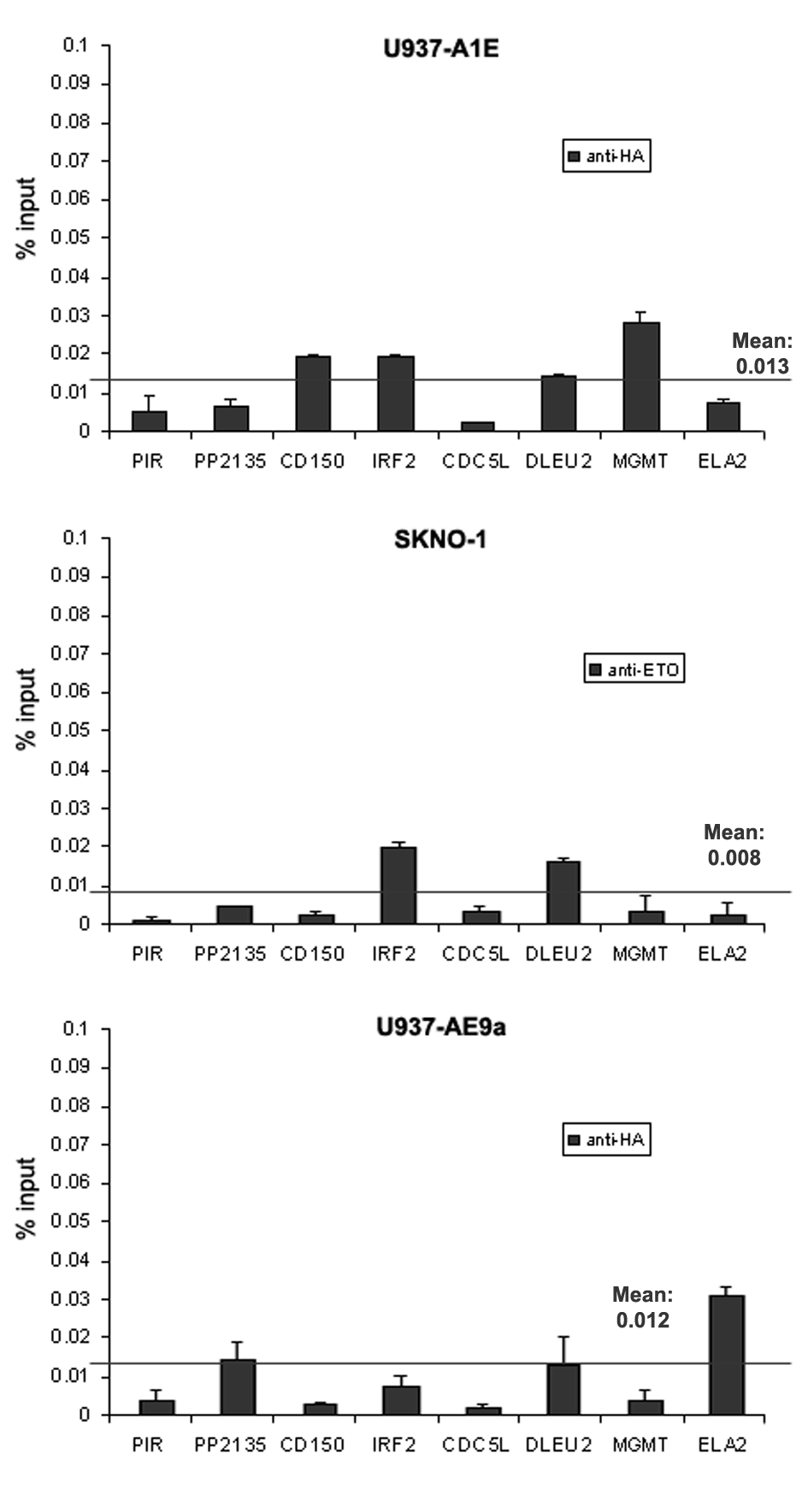


**Figure S1. Calculation of baseline values for qChIP experiments.** qChIP was performed as described in Materials and Methods using an anti-HA (U937-AE and U937T-AE9a cells) or an anti-ETO antibody (SKNO-1) on the promoter regions of 8 genes that did not display significant AML1/ETO enrichment according to ChIP-chip data. Mean baseline enrichment values were calculated, and are displayed as black vertical lines in Figure 1 and Figure S2. Oligonucleotide sequences used for qPCR are reported in Supplementary Methods.
