# Supplementary material for: AML1/ETO Oncoprotein Is Directed to AML1 Binding Regions and Co-Localizes with AML1 and HEB on Its Targets
Source: PLoS Genet. 2008 Nov 28;4(11):e1000275. doi: 10.1371/journal.pgen.1000275 (PMC2577924; doi:10.1371/journal.pgen.1000275)
Supplement: Figure S2 — qChIP analysis of AML1/ETO9a binding on AML1/ETO target regions. (0.18 MB DOC) [file pgen.1000275.s012.doc]

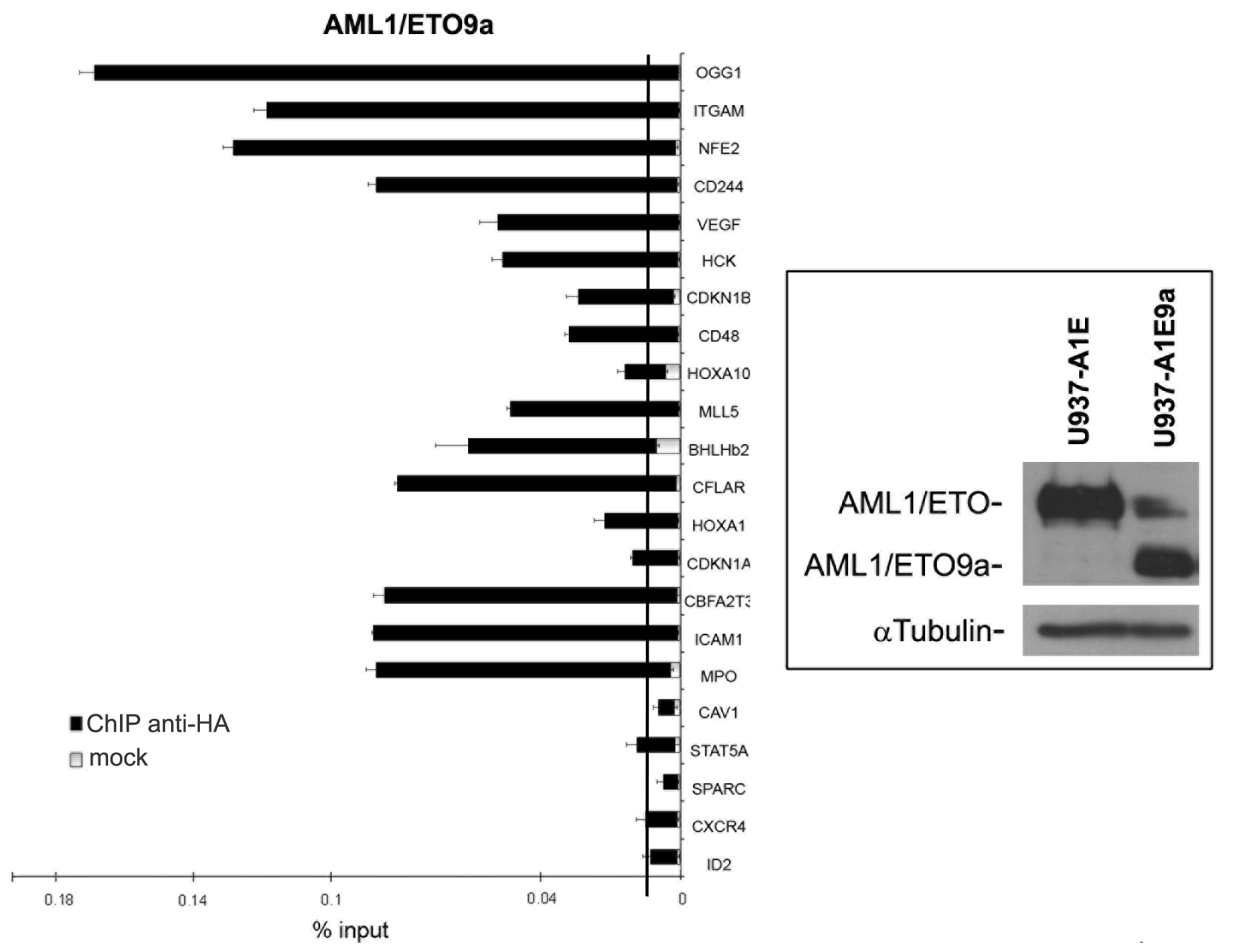


**Figure S2. qChIP analysis of AML1/ETO9a binding on AML1/ETO target regions.** qPCR was performed on chromatin from U937T-AE9a cells, using an anti-HA antibody. Black bars represent binding of AML1/ETO9a on 22 promoters identified as binding regions for full-length AML1/ETO (Figure 1). The light grey portion on each bar represents the level of enrichment obtained in the mock experiment (without antibody). The baseline (represented as a vertical black line) corresponds to the mean level of enrichment obtained by qChIP on 8 negative control genes. The leukemogenic AML1/ETO9a isoform displays a binding pattern that is similar to that of AML1/ETO, although enrichment values differ. Protein levels of AML1/ETO and AML1/ETO9a in the corresponding U937 cell lines were analyzed by Western blotting with an anti-HA antibody.
