# Supplementary material for: AML1/ETO Oncoprotein Is Directed to AML1 Binding Regions and Co-Localizes with AML1 and HEB on Its Targets
Source: PLoS Genet. 2008 Nov 28;4(11):e1000275. doi: 10.1371/journal.pgen.1000275 (PMC2577924; doi:10.1371/journal.pgen.1000275)
Supplement: Figure S3 — Validation of transcriptional regulation of AML1/ETO target genes identified by gene expression profiling. (0.17 MB DOC) [file pgen.1000275.s013.doc]

**
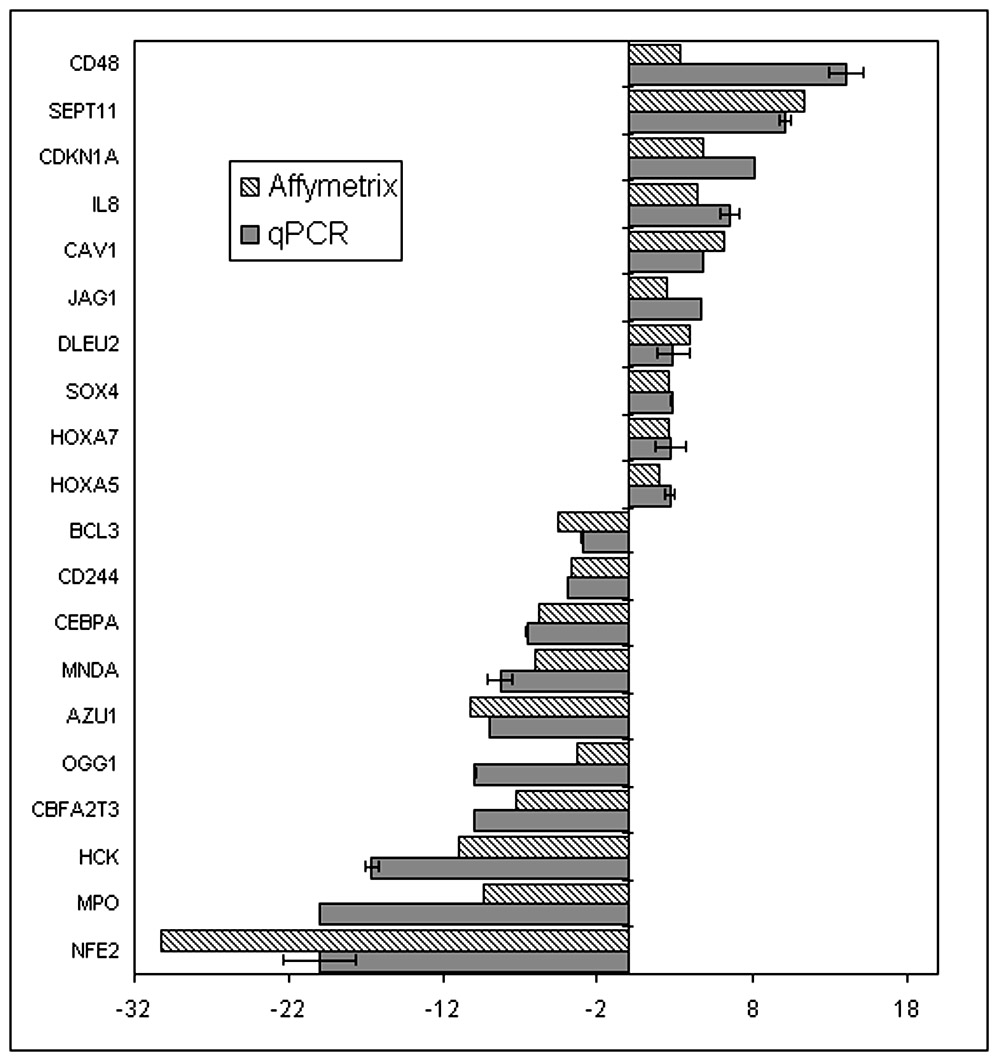
**

**Figure S3.** **Validation of transcriptional regulation of AML1/ETO target genes identified by gene expression profiling.** Histogram of microarray and qPCR results for 20 validated genes. Complete results are reported in Table S3.
