# Supplementary material for: AML1/ETO Oncoprotein Is Directed to AML1 Binding Regions and Co-Localizes with AML1 and HEB on Its Targets
Source: PLoS Genet. 2008 Nov 28;4(11):e1000275. doi: 10.1371/journal.pgen.1000275 (PMC2577924; doi:10.1371/journal.pgen.1000275)
Supplement: Figure S4 — AML1/ETO interacts with the E-protein HEB in U937-AE cells. (0.10 MB DOC) [file pgen.1000275.s014.doc]

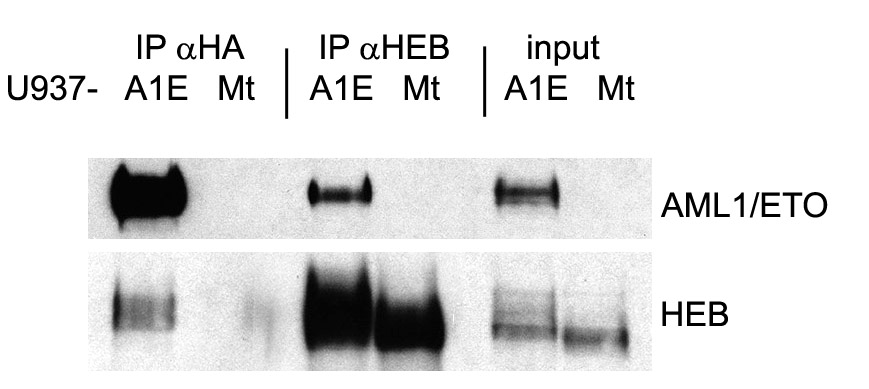


**Figure S4**: **AML1/ETO interacts with the E-protein HEB in U937-AE cells.** Co-immunoprecipitation experiments were performed using the anti-HA and anti-HEB antibodies in cell lysates from U937-AE and U937-Mt cells, as indicated above the blots. Western blots were decorated with the anti-HA (top panel) and anti-HEB (bottom panel) antibodies.
