# Supplementary material for: AML1/ETO Oncoprotein Is Directed to AML1 Binding Regions and Co-Localizes with AML1 and HEB on Its Targets
Source: PLoS Genet. 2008 Nov 28;4(11):e1000275. doi: 10.1371/journal.pgen.1000275 (PMC2577924; doi:10.1371/journal.pgen.1000275)
Supplement: Figure S5 — qChIP analysis of HEB binding on AML1/ETO target regions in SKNO-1 cells. (0.12 MB DOC) [file pgen.1000275.s015.doc]

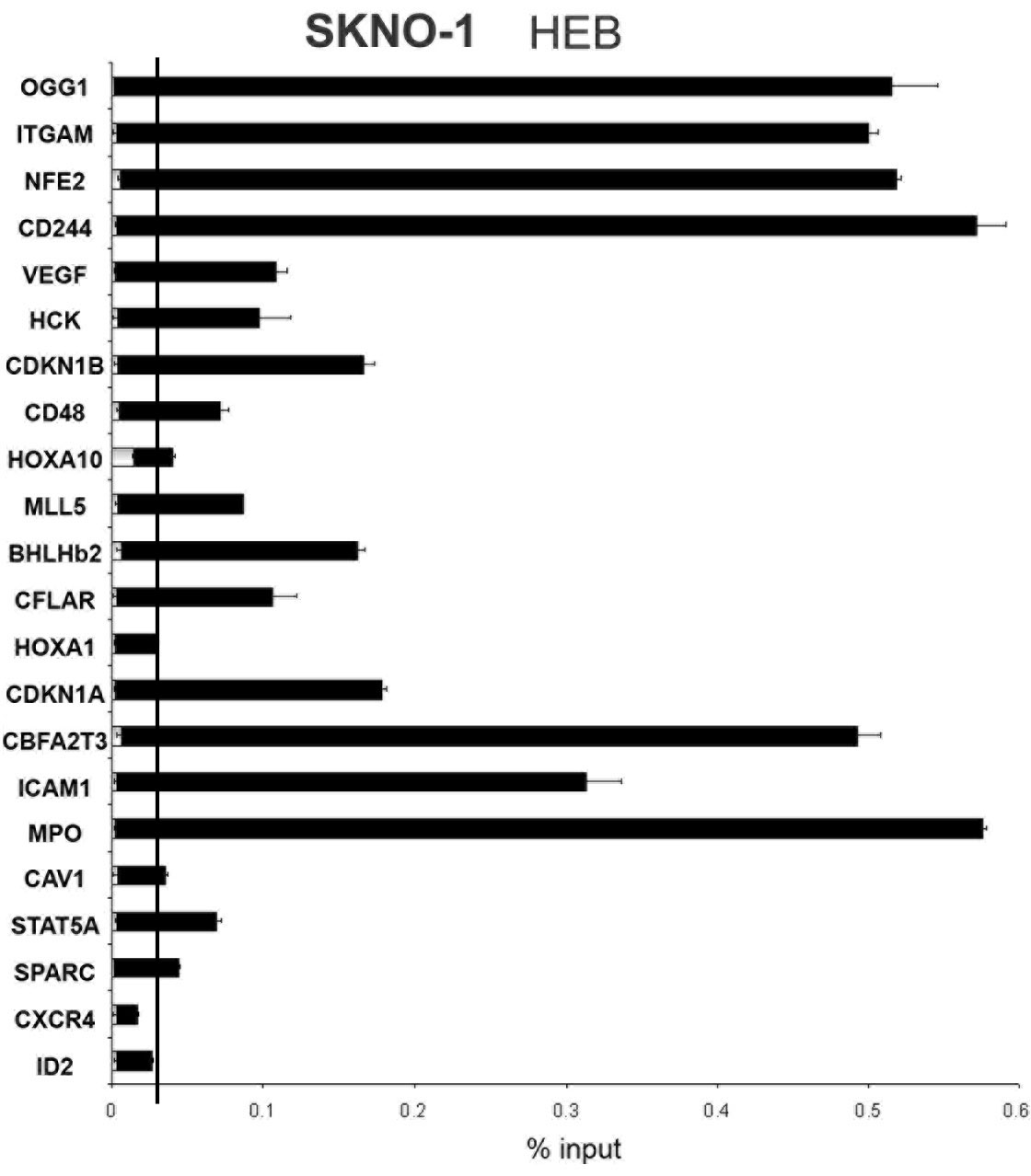


**Figure S5**: **qChIP analysis of HEB binding on AML1/ETO target regions in SKNO-1 cells.** qPCR was performed on the ChIP products obtained from SKNO-1 cells using an anti-HEB antibody on 22 promoters identified as binding regions for full-length AML1/ETO. Levels of enrichment obtained in the mock experiment (without antibody) are represented in grey. The baseline (represented as a vertical black line) corresponds to the mean level of enrichment obtained by qChIP on the eight genes that do not present AML1/ETO binding described in figure S1. Comparison of this panel with the corresponding qChIP analysis for AML1-ETO (Figure 1) reveals correlation between absolute levels of enrichment for the two transcription factors.
