# Supplementary material for: AML1/ETO Oncoprotein Is Directed to AML1 Binding Regions and Co-Localizes with AML1 and HEB on Its Targets
Source: PLoS Genet. 2008 Nov 28;4(11):e1000275. doi: 10.1371/journal.pgen.1000275 (PMC2577924; doi:10.1371/journal.pgen.1000275)
Supplement: Figure S6 — Rearrangement of HEB and AML1 binding patterns in AML1/ETO expressing cells. (0.88 MB DOC) [file pgen.1000275.s016.doc]

**
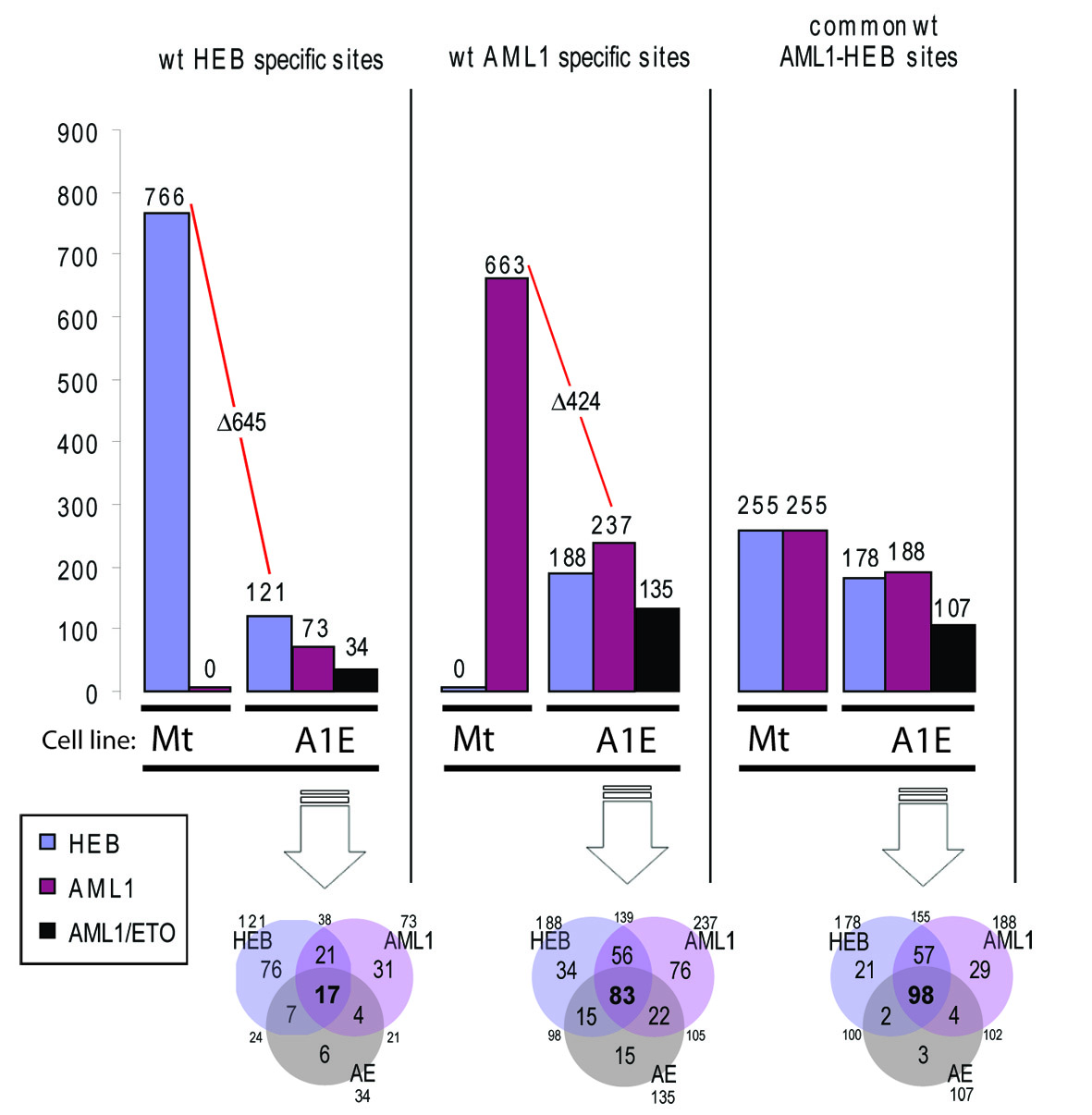
**

**Figure S6**: **Rearrangement of HEB and AML1 binding patterns in AML1/ETO expressing cells.** Histograms represent the number of binding sites for each transcription factor in U937-Mt and U937-AE cells (the number above each bar indicates the number of binding sites; the cell line is indicated under the graph). The left panel refers to the 766 HEB-specific binding sites in U937-Mt cells, the middle panel to the 663 AML1 specific binding sites and the right panel to the 255 sites shared by AML1 and HEB. Each panel shows the redistribution of transcription factors within these sites in U937-AE cells. Loss of HEB and AML1 binding at several specific sites in AML1/ETO expressing cells is reported by diagonal red lines (∆645 and ∆424, respectively). Binding regions that are common for HEB and AML1 in U937-Mt cells (right panel) are frequent sites of co-localization with AML1/ETO, and both transcription factors are less prone to be displaced upon fusion protein expression. The Venn diagrams at the bottom of each panel specify, for each category, the number of overlapping binding sites for the three transcription factors in U937-AE cells.
