# Supplementary material for: AML1/ETO Oncoprotein Is Directed to AML1 Binding Regions and Co-Localizes with AML1 and HEB on Its Targets
Source: PLoS Genet. 2008 Nov 28;4(11):e1000275. doi: 10.1371/journal.pgen.1000275 (PMC2577924; doi:10.1371/journal.pgen.1000275)
Supplement: Figure S7 — AML1/ETO preferentially binds in the proximity of expressed genes. (0.10 MB DOC) [file pgen.1000275.s017.doc]

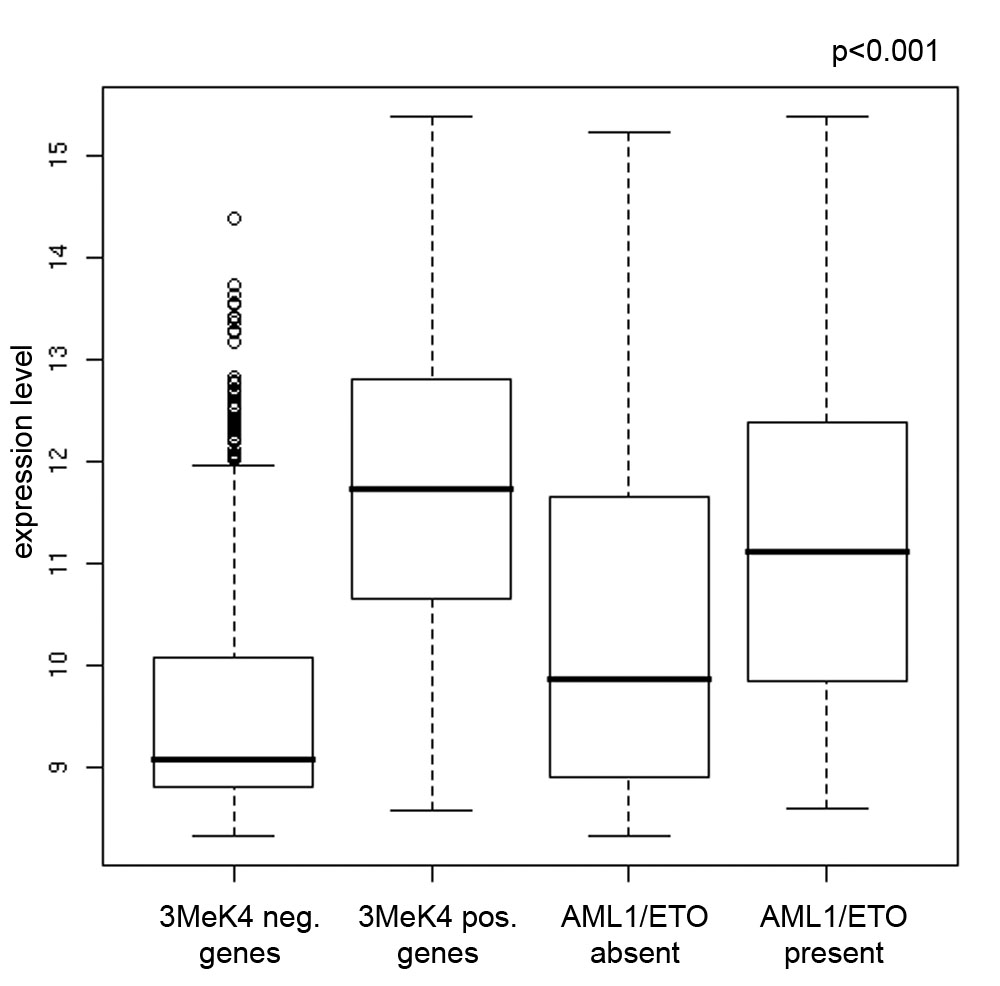


**Figure S7**: **AML1/ETO preferentially binds in the proximity of expressed genes.** Box plot representation of the expression values of genes associated to tri-methylation of lysine 4 on histone 3 (H3K4me3 pos genes) compared to the expression values of all other genes on chromosome 19 (H3K4me3 neg genes) clearly shows that this histone modification is associated to higher expression levels. The median expression value is indicated by a black line within the box. Genes associated to AML1/ETO binding regions (AML1/ETO present) display a significantly higher median expression value than other genes on the array (AML1/ETO absent).
