# Supplementary material for: AML1/ETO Oncoprotein Is Directed to AML1 Binding Regions and Co-Localizes with AML1 and HEB on Its Targets
Source: PLoS Genet. 2008 Nov 28;4(11):e1000275. doi: 10.1371/journal.pgen.1000275 (PMC2577924; doi:10.1371/journal.pgen.1000275)
Supplement: Figure S8 — Displacement of HEB from its native binding sites is associated to transcriptional regulation. (0.20 MB DOC) [file pgen.1000275.s018.doc]

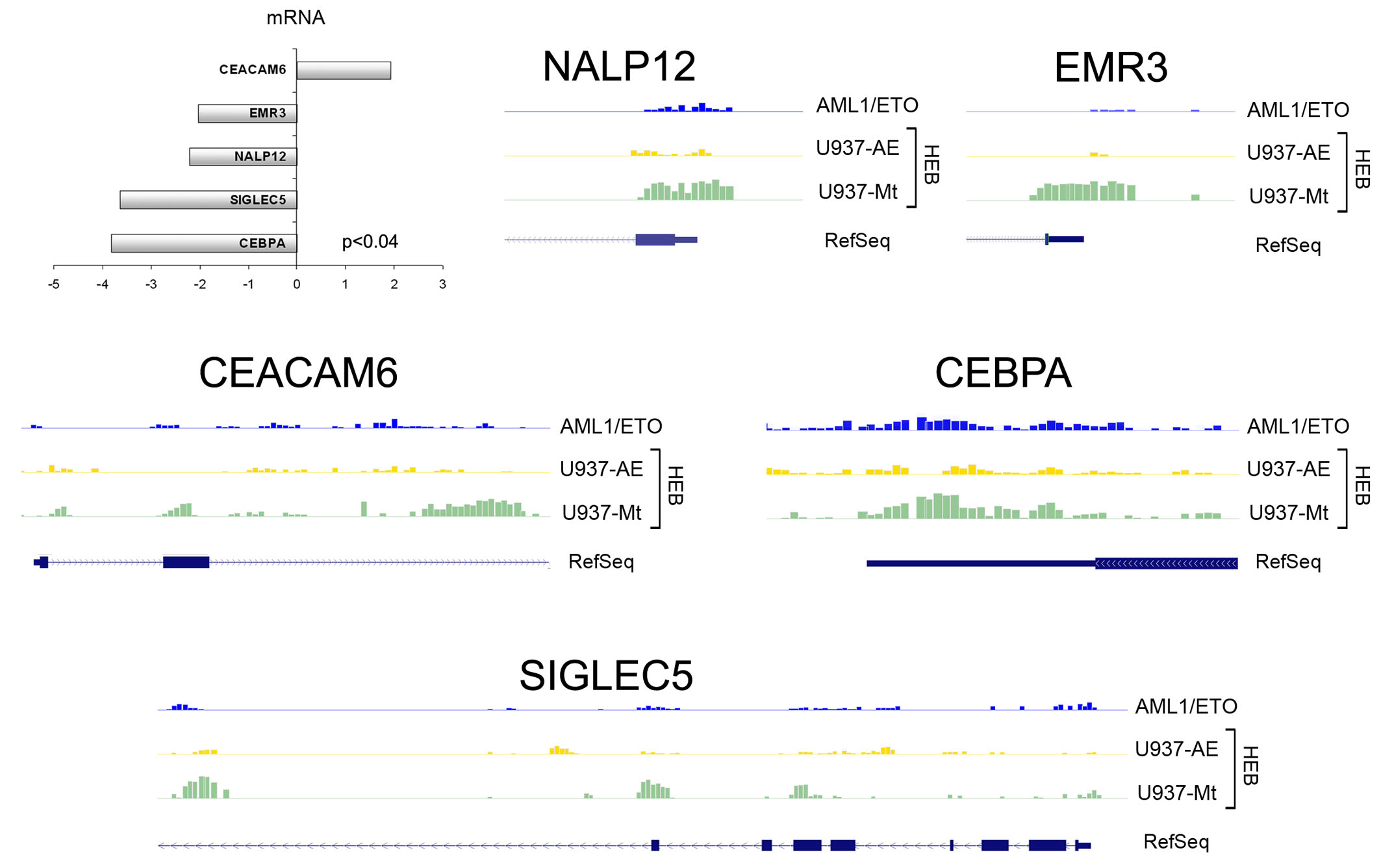


**Figure S8:** **Displacement of HEB from its native binding sites is associated to transcriptional regulation.** The binding profiles of 5 genes on chromosome 19 (*NALP12, EMR3, CEACAM6, CEBPA, SIGLEC5*) that are regulated by AML1/ETO without direct binding are shown. Fold change of mRNA levels in U937-AE compared to U937-Mt, as derived from expression tiling data, are shown in the upper-left histogram. Screenshots of the gene loci show the absence of AML1/ETO binding (top lane), and the pattern of HEB binding in U937-AE and U937-Mt cells (2^nd^ and bottom lane, respectively). In all cases, HEB binding was detected only in control cells and not in AML1/ETO expressing cells. Below each panel is a schematic representation of the locus.
