# Supplementary material for: AML1/ETO Oncoprotein Is Directed to AML1 Binding Regions and Co-Localizes with AML1 and HEB on Its Targets
Source: PLoS Genet. 2008 Nov 28;4(11):e1000275. doi: 10.1371/journal.pgen.1000275 (PMC2577924; doi:10.1371/journal.pgen.1000275)
Supplement: Text S1 — Supplementary data and methods. (1.58 MB DOC) [file pgen.1000275.s019.doc]

**Text S1. Supplementary Data and Methods**

**Supplementary Methods**

- ChIP-chip data analysis
- qChIP validation of ChIP-chip results
- Affymetrix GeneChip hybridization
- Affymetrix data analysis
- qRT-PCR validation of expression profiling
- Expression tiling array: experimental procedures and data analysis

**Supplementary Data**

- Sequence analysis of AML1/ETO, AML1 and HEB binding regions
- Correlation between AML1, HEB and AML1/ETO binding profiles and gene expression
- **Figure S9:** Sequence analysis of AML1/ETO binding regions.
- **Figure S10**: Analysis of AML1 motif in the sequences corresponding to 408 AML1/ETO peaks on chromosome 19.
- **Figure S11**: Unsupervised sequence analysis of AML1/ETO and AML1 ChIP-chip data by MatrixREDUCE algorithm.
- **Figure S12**: HEB binds to regions containing an AML1 consensus only in AML1/ETO expressing cells.
- **Table S11.** Sequence analysis of AML1/ETO peaks at regulated genes. Full list of significantly enriched matrices is reported.

**Supplementary Methods**

**ChIP-chip data analysis**

PeakPicker (script available upon request) centers a sliding window of user-defined size around every probe of the array, then picks out the number of probes within the window that are above a given percentile and compares it to the expected number, calculating a Chi-square and its relative p-value. A list of probe-associated p-values is generated and used by PeakPicker to extract regions where p-values are contiguously significant and thus are very likely to represent a real enrichment of the original ChIP product (minimum number of values: 3; maximum distance: 100bp). The minimum requirement for a region to be selected is to retain at least 3 probes that passed the percentile selection.

The strength of this analysis relies on the p-value assignment, which takes into account the environment of every probe and, therefore: i) prevents underestimation of signals coming from regions poorly represented on the array (i.e. because of the exclusion of highly repetitive sequences); ii) reduces the risk of splitting into two or more peaks a contiguous binding region.

For the analysis of Promoter Array data, since probes are more sparse (110 bp interval), a lower stringency analysis was used: 90^th^ percentile, window of 1000 bp, |log(p-value)|>2. We then selected peaks present in both biological replicates, and not retrieved in a parallel analysis of the control cell line U937-Mt.

For the analysis of Chr.19 Array data, stringent parameters were applied: 98^th^ percentile, window of 500 bp, |log(p-value)|>7. For the identification of 408 peaks of AML1/ETO, two separate lists deriving from anti-HA and anti-ETO experiments were compiled, and peaks retrieved in the mock cell line U937-Mt were eliminated (roughly 3% of peaks). Next, the two lists were compared and common regions with at least 60% of physical overlap were selected.

**qChIP validation of ChIP-chip results**

The following oligonucleotides were used to validate 22 AML1/ETO binding regions, as described in Materials and Methods:

| BHLHb2 | for | CTGAAGCTGCATCTCAAAGC |
| --- | --- | --- |
| BHLHb2 | rev | AGGACTGCCCTGGAGAGG |
| CBFA2T3 | for | CAGTTGGGCTGGAAACTGAC |
| CBFA2T3 | rev | GAGCTCAGAAGTGGGGACTG |
| CD244 | for | AGTTTGGCTTGTGGGAACTG |
| CD244 | rev | GACAAGGCCACTGAGAAAGC |
| CD48 | for | TTGCTGGGAAGTTCTGGAAG |
| CD48 | rev | CAGACACGAATCCCAACCTC |
| CFLAR | for | GCGGTTATTTTCCAGAGCAG |
| CFLAR | rev | CAAGCTTTCCTTGCACTTGG |
| CXCR4-1f | for | GGAAAAGATGGGGAGGAGAG |
| CXCR4-1r | rev | AGTGGGCTAAGGGCACAAG |
| HCK | for | GGGTTAGTATCTTGGGAGC |
| HCK | rev | CCCACTCCTAGGGATTCG |
| CAV1 | for | CTCTACACCGTTCCCATCC |
| CAV1 | rev | CTTGCTTCTCGCTCAGCTC |
| HOXA1 | for | TGGGGTATTCCAGGAAGGAG |
| HOXA1 | rev | GCAGGACCAGGTCACTCAG |
| HOXA10 | for | CTCCGAGCATGACATTGTTG |
| HOXA10 | rev | CCAGAAAGGGCTATCTGCTC |
| ICAM1 | for | CAAGGCCGAAAGGGGAAGC |
| ICAM1 | rev | CGGCGTCCTCTCTCTACAC |
| ID2 | for | ATAGCCCTGCCTCCTTCTTG |
| ID2 | rev | GCTAAGGAGCTCTCACAGG |
| ITGAM | for | GCTTCCTTGTGGTTCCTCAG |
| ITGAM | rev | AGGAGCCAGAACCTGGAAG |
| MLL5 | for | CCTCCTTGACGTTGTACCC |
| MLL5 | rev | GAGTCCTTGAGGGCCTCTG |
| MPO | for | CACTGATCCTGTCCCAGAG |
| MPO | rev | AGGAACCAGGAGAGAGCAG |
| NFE2 | for | GGTTAGCAGCATACGTGGAG |
| NFE2 | rev | ACGATACGGAGAAAACCACG |
| OGG1 | for | CCACCCTGATTTCTCATTGG |
| OGG1 | rev | CAACCACCGCTCATTTCAC |
| CDKN1A | for | GGCGGTTGTATATCAGGGC |
| CDKN1A | rev | GGCTCCACAAGGAACTGAC |
| CDKN1B | for | AGAACGGAAGGTTGGTTTCC |
| CDKN1B | rev | CCCATCATCTTGGTTTGAGC |
| SPARC | for | GGTTTCCTGTTGCCTGTCTC |
| SPARC | rev | GGGGTCACACATACCTCAG |
| STAT5A | for | TTCCTTTCCTCGTGATCCTG |
| STAT5A | rev | TGCAGAAGCCAAAATGTCTG |
| VEGF | for | GGTTTGGATCCTCCCATTTC |
| VEGF | rev | CAGTCAGTGGTGGGGAGAG |

A baseline for AML1/ETO enrichment was calculated by qChIP in 8 negative promoters, using the following oligonucleotides:

| PIR | for | GGAGGAAAATGGAATTCGTG |
| --- | --- | --- |
| PIR | rev | AAGCCTTCCCTAAATGATTCC |
| PP2135 | for | GTTTGGGAATGTGGGAAATG |
| PP2136 | rev | ACAAGAGCACCACATTGCAG |
| CD150 | for | GTTTTGGCCGAGGTGACTAC |
| CD151 | rev | CAAGAAGCCAGGCACTTAGC |
| IRF2 | for | TACCTTGCGGAATTGTATTGG |
| IRF3 | rev | CATGCCCTCGAGAGAAACAC |
| CDC5L | for | AAATAGGGCATTTCCCAACC |
| CDC5L | rev | GAACATCTCTGGCTCCATCC |
| DLEU2 | for | CTTCTTCGGTTGCAGTCCTC |
| DLEU3 | rev | GCTTTGCAAAAGCGCTACAC |
| MGMT | for | TCTTCGGGATTTATTGTCTGTGG |
| MGMT | rev | CCGAAGGAAGCTGACCTGG |
| ELA2 | for | AGCCAATCCAGCGTCTTGTC |
| ELA3 | rev | CCTCCACTTCCTCTCCCCTG |

Analysis of promotorial and intragenic binding regions of AML1/ETO was performed with the following oligonucleotides:

| BCL3-A | for | AAGATCTCTGCGCCTGTCTC |
| --- | --- | --- |
| BCL3-A | rev | TGCTCGGAAAATCCTAGGTG |
| BCL3-B | for | GTCAGCACCCGTCACTCAC |
| BCL3-B | rev | GGGTTAAGGTTGGAGGAAGC |
| GNA15-A | for | GTCTCCCAGTTTTGCTGGTC |
| GNA15-A | rev | GTGCAGATCTTCCAGGATGG |
| GNA15-B | for | ACATAGCTGGCTCCAGTTGC |
| GNA15-B | rev | AGGAGGACAGGAGGAAGTG |
| GNA15-C | for | GTCCAAAGCCTCTGTTCTGG |
| GNA15-C | rev | TCGCTGATAACCATCTGTGC |
| UHRF1-A | for | CTCTCCCGGAAGGAGAAGTC |
| UHRF1-A | rev | CCTCCCTTTGTTCCTTCCTC |
| UHRF1-B | for | CTCTCCCTCCGTCTGTCTTG |
| UHRF1-B | rev | AACTGCTCTGCCTTCCTGAG |
| VAV1-A | for | TGGTGTCCTTCTGTGTCAGC |
| VAV1-A | rev | GACCTCACGCAGGTTGATG |
| VAV1-B | for | TAACAGGCATTTCCGGTTTC |
| VAV1-B | rev | ACGGCAGCAATCAACTCC |

**Affymetrix GeneChip hybridization**

All steps of the labeling protocol were performed following indications suggested by Affymetrix (for a detailed protocol see: <http://services.ifom-ieo-campus.it/Affymetrix/PDF/Protocol_190406.pdf>). Each biotin-labeled target was hybridized to two copies of the Affymetrix HG-U133 Plus 2.0 array (Affymetrix, USA). Hybridizations were performed for 14-16 hours at 45°C in a rotisserie oven. GeneChip cartridges were washed and stained in the Affymetrix fluidics station following the EukGE-WS2 standard protocol. Images were scanned using an Affymetrix GeneArray Scanner, using default parameters.

**Affymetrix data analysis**

“Absolute analysis” was performed for each chip with GCOS software using default parameters. Performance of labeled targets was evaluated on the basis of scaling factor, background and noise values, % present calls and average signal value. Results derived from U937-AE cells (sample) were compared to results from U937-Mt cells (reference) by “comparative analysis” with GCOS, using the reference chips as baseline. Each sample chip was compared to both reference chips and duplicate sample and reference chips were compared to each other for calculation of noise. Data were then analyzed using GenePicker software [1], by combining the Change-Fold Change and the t-test analysis, as previously described [2]. Cut-off value for the Change-Fold Change analysis was >1.5, whereas t-test parameters were: p-value < 0.05 and fold change >2. Regulated genes were then annotated using batch SOURCE (http://genome-www5.stanford.edu/cgi-bin/source/sourceSearch), starting from the accession number associated to the corresponding Affymetrix Probe Set.

**qRT-PCR validation of expression profiling**

Reactions were performed as described in Materials and Methods. Each sample was run in triplicate. The mean value of the replicates for each sample was calculated and expressed as cycle threshold (*C_T_*, cycle number at which each PCR reaction reaches a predetermined fluorescence threshold, set within the linear range of all reactions). The amount of gene expression was then calculated as the difference (Δ*C_T_*) between the *C_T_* value of the sample for the target gene and the mean *C_T_* value of that sample for the endogenous control (*GAPDH*). Relative expression was calculated as the difference (ΔΔ*C_T_*) between the Δ*C_T_* values of the AML1/ETO sample and of the control sample, Mt, for each target gene. See Table S3 for the full list of oligonucleotides used in qRT-PCR analysis.

For analysis of HEB expression we used the following primers:

HEB for CGTGGCAGTCATCCTTAGTC

HEB rev TGCTTTGGGGTTAAGGTTCC

**Expression tiling array: experimental procedures and data analysis**

Total RNA from U937-AE and Mt cells was extracted and treated with RNAse-free DNAse (Qiagen, USA). First strand cDNA synthesis was performed on 15μg of total RNA with Superscript III (Invitrogen, USA) using Oligo(dT)_12–18_ Primer according to manufacturer’s protocol. Complementary second strand was then synthesized in a reaction containing 200μM dNTP mix, 10 U E.Coli DNA Ligase (NEB, USA), 40 U E.Coli DNA Polymerase I (NEB, USA), 2 U E.Coli RNAseH and 1X Second-Strand Reaction buffer (Invitrogen, USA). Reaction was performed at 16°C for 2 hours, double-stranded cDNA (ds-cDNA) was purified by phenol/chlorophorm extraction followed by ethanol precipitation and finally treated with DNAse-free RNAse (Roche, USA) to eliminate residual carryover of rRNA. A second round of purification was performed and 4 μg of cDNA were labeled by NimbleGen Services using random priming and Cy3 fluorescent dye. Two independent cDNA samples from every cell line (U937-AE and Mt) were hybridized to Chr.19 arrays.

This approach differs from ChIP-chip in that the hybridization is performed with a single sample, without references. Therefore, the output file contains raw fluorescence values instead of logarithmic ratios. Mean fluorescence of every probe is calculated from the two replicates. Dataset from the two cell lines were then analyzed using the perl script TEA (M. Cesaroni et al., manuscript in preparation) which calculates Log2 of the mean value of probes matching the coding region of any annotated gene. Using Hg.18 REFSEQ tables from UCSC, 1,668 transcribed sequences corresponding to 1,305 non-redundant genes were annotated on chromosome 19. The log2|mean value| was then calculated using all the probes matching to a transcript. Finally, a fold change between the two cell lines was calculated, a t-test was perfomed and the associated p-value was derived and reported. A threshold of |FC| >1.5, p-value<0.05 was used for the identification of regulated genes.

**References**

1. Finocchiaro G, Parise P, Minardi SP, Alcalay M, Muller H (2004) GenePicker: replicate analysis of Affymetrix gene expression microarrays. Bioinformatics 20: 3670-3672.

2. Alcalay M, Meani N, Gelmetti V, Fantozzi A, Fagioli M, et al. (2003) Acute myeloid leukemia fusion proteins deregulate genes involved in stem cell maintenance and DNA repair. J Clin Invest 112: 1751-1761.

**Supplementary Data**

**Sequence analysis of AML1/ETO, AML1 and HEB binding regions**

Sequence analysis of the DNA regions bound by a transcription factor can be performed through bioinformatics approaches that yield different kinds of information. Supervised approaches search for the presence of defined matrices within a group of sequences, and highlight significant enrichments using a random set of sequences as control. The main limitations of these methods are that they only explore a defined set of matrices and they require previous manipulations of raw data to identify DNA sequences bound by a transcription factor. Unsupervised prediction methods are extremely powerful since they do not rely on predetermined matrices and do not require an arbitrary definition of specifically bound regions.

DNA sequence determinants associated to AML1/ETO binding on the promoter array and on the Chr. 19 array were analyzed using both approaches. Peaks associated to the 358 putative direct target genes identified in the promoter-biased approach were first analyzed through a motif search using the CLOVER algorithm (Cis-eLement OVERrepresentation) [1], and interrogating two different collections of transcription factor biding sites (TFBS): the JASPAR CORE [2] and the TRANSFAC database [3]. First, nucleotide sequences were extracted using the University of California Santa Cruz (UCSC) genome browser (UCSC human database release hg17, May 2004). Two subsets of sequences associated with Affymetrix up- and down-regulated genes were individually scanned for 585 vertebrate position weight matrices from the professional TRANSFAC10.4 database and for the 123 matrices from the JASPAR CORE database. CLOVER parameters were set to 1,000 randomizations and a p-value threshold of 0.05. For estimation of p-values, we supplied as background the “upstream5000” dataset, derived from the UCSC human database, which includes 5000 bp upstream of annotated TSS of RefSeq genes.

Three groups of enriched motifs were identified: one was common to the promoter regions of both up- and down-regulated genes; the second was specific to the promoters of downregulated genes, and the third specific to upregulated genes (Figure S9A and Table S11). The AML1 core sequence (Transfac matrix: M00722) was significantly enriched only in the peaks corresponding to the promoters of downregulated genes that also displayed overrepresentation of other transcription factor binding sites (TFBS) related to hematopoietic differentiation, such as VDR and IK3 (Figure S9). Conversely, peaks from the promoters of upregulated genes were characterized by an overrepresentation of TFBS for three different members of the forkhead family of transcription factors (FOXO1, FOXO3 and FOXO4). These data suggest that AML1/ETO-dependent transcriptional activation may rely on transcriptional complexes that include the fusion protein, but depend on other factors for their DNA binding properties.

The same approach was used to analyze sequences bound by AML1/ETO on the Chr.19 Array, interrogating the TRANSFAC database to study the 408 AML1/ETO peaks. Strikingly, 346 peaks (85%) contained an AML1 binding site. No significant differences in TFBS enrichment was found by comparing peaks lying in the promoters from those in the gene body (Figure S10).

Analyses that rely on fixed matrices may miss binding sites that do not perfectly reflect any known sequence consensus. The MatrixREDUCE algorithm [4], which infers sequence specificity of a transcription factor from raw ChIP-chip data without using pre-existing TFBS matrices, was therefore used. All probe sequences of Promoter Arrays or Chr.19 Arrays and corresponding signals of log2 transformed ChIP-chip ratios were used. The parameters for all MatrixREDUCE analyses were the following: length of each of the two dyads of the seed motifs = 3, length of the added flanks on each side = 3, minimum gap = 0, k cross-validations = 2, maximum acceptable *P*-value = 10^-3^ and maximum dyad gap = 10. The best 20 fit position specific affinity matrix (PSAM) matrices obtained were then compared with JASPAR and TRANSFAC databases using STAMP algorithm to recognize potential similarity with known TFBS [5]. In STAMP, Pearson correlation coefficient was used as column comparison metric, ungapped Smith-Waterman as alignment method, iterative refinement as multiple alignment strategy and UPGMA as tree-building algorithm on untrimmed PSAMs.

Analysis of the AML1/ETO binding pattern on human promoters resulted in the identification of one PSAM corresponding to the AML1 consensus sequence (Figure S9B). Using this approach on the Chr. 19 Array datasets, a subset of significant PSAMs associated to AML1/ETO binding was inferred. In particular, four matrices were identified, which corresponded with high stringency to the known binding sites for AML1, AP.1, Ets-1 and the E protein HEB (Figure S11). Notably, the same PSAMs were also retrieved from AML1 ChIP-chip datasets deriving from both U937-AE and U937-Mt cells. These results confirm that there is a specific sequence signature associated to genomic occupancy of AML1/ETO, which is common to AML1. The fact that this signature was only identified in the Chr.19 array may reflect its high density of tiling, as opposed to the more sparse location of oligonucleotides on the Promoter Array, and is not correlated to the location of TFBS with respect to gene structure (Figure S10).

MatrixREDUCE analysis of HEB ChIP-chip data identified a specific E-box motif corresponding to the HEB consensus in both U937-AE and U937-Mt cells, but the AML1 motif was inferred only in U937-AE (Figure S12). This result demonstrates that in the presence of AML1/ETO, HEB binds to specific sites on the genome, which are enriched for the AML1 consensus sequence.

**Correlation between AML1, HEB and AML1/ETO binding profiles and gene expression**

Our data show that AML1/ETO often co-localizes with AML1 on DNA and causes a redistribution of HEB binding pattern. The correlation in DNA occupancy of these three transcription factors was, therefore, analyzed. In the absence of AML1/ETO (U937-Mt cells), there are 255 common binding regions for HEB and AML1 on chromosome 19 (Figure S6). AML1/ETO displaces HEB primarily from its “unique” binding locations (84% or 645/766, of HEB peaks disappear in U937-AE), and has less impact on HEB binding where it co-localizes to AML1 (30%, or 76/255, displacement) (Figure S6). To a lesser extent, a similar pattern is observed for AML1 binding: 64% (424/663) of “unique” AML1 binding sites identified in U937-Mt disappear in the presence of AML1/ETO, whereas there is displacement of AML1 in 26% of DNA regions that also bind HEB (Figure S6).

These data show that in U937-Mt cells, HEB and AML1 localize to the same genomic regions in approximately 25% of their global binding sites, suggesting they may be involved in co-regulation of common target genes. Furthermore, the binding of both transcription factors to these common regions is less affected by AML1/ETO expression than in other genomic locations.

**References**

1. Frith MC, Fu Y, Yu L, Chen JF, Hansen U, et al. (2004) Detection of functional DNA motifs via statistical over-representation. Nucleic Acids Res 32: 1372-1381.

2. Sandelin A, Alkema W, Engstrom P, Wasserman WW, Lenhard B (2004) JASPAR: an open-access database for eukaryotic transcription factor binding profiles. Nucleic Acids Res 32: D91-94.

3. Wingender E, Dietze P, Karas H, Knuppel R (1996) TRANSFAC: a database on transcription factors and their DNA binding sites. Nucleic Acids Res 24: 238-241.

4. Foat BC, Houshmandi SS, Olivas WM, Bussemaker HJ (2005) Profiling condition-specific, genome-wide regulation of mRNA stability in yeast. Proc Natl Acad Sci U S A 102: 17675-17680.

5. Mahony S, Benos PV (2007) STAMP: a web tool for exploring DNA-binding motif similarities. Nucleic Acids Res 35: W253-258.


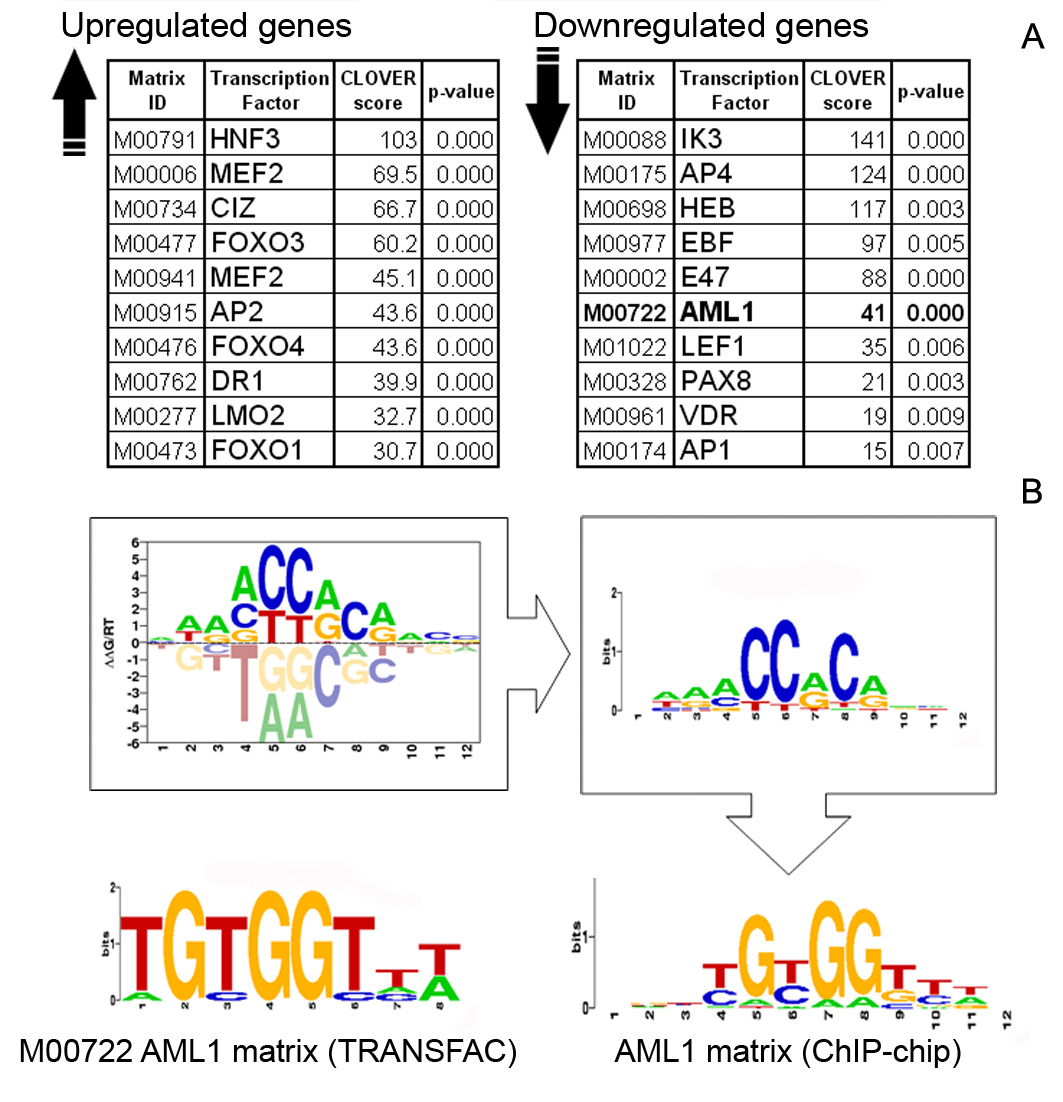


**Figure S9: Sequence analysis of AML1/ETO binding regions.**

**A.** Enriched TRANSFAC matrices derived from motif analysis of AML1/ETO target regions reveal distinct signatures in upregulated (left) and downregulated (right) genes. Only the latter group shows enrichment for AML1 binding sites (bold). The full list of enriched matrices is available in Table S11.

**B.** ChIP-chip data from U937-AE cells define *de novo* an AML1 affinity matrix, suggesting that most of AML1/ETO binding across human promoters is driven by AML1 motif recognition. Upper left panel shows the PSAM inferred by MatrixREDUCE, which is transformed into a positional weight matrix PWM (upper right). The reverse complement PWM (bottom right) strongly resembles the TRANSFAC AML1 matrix M00722 (bottom left).


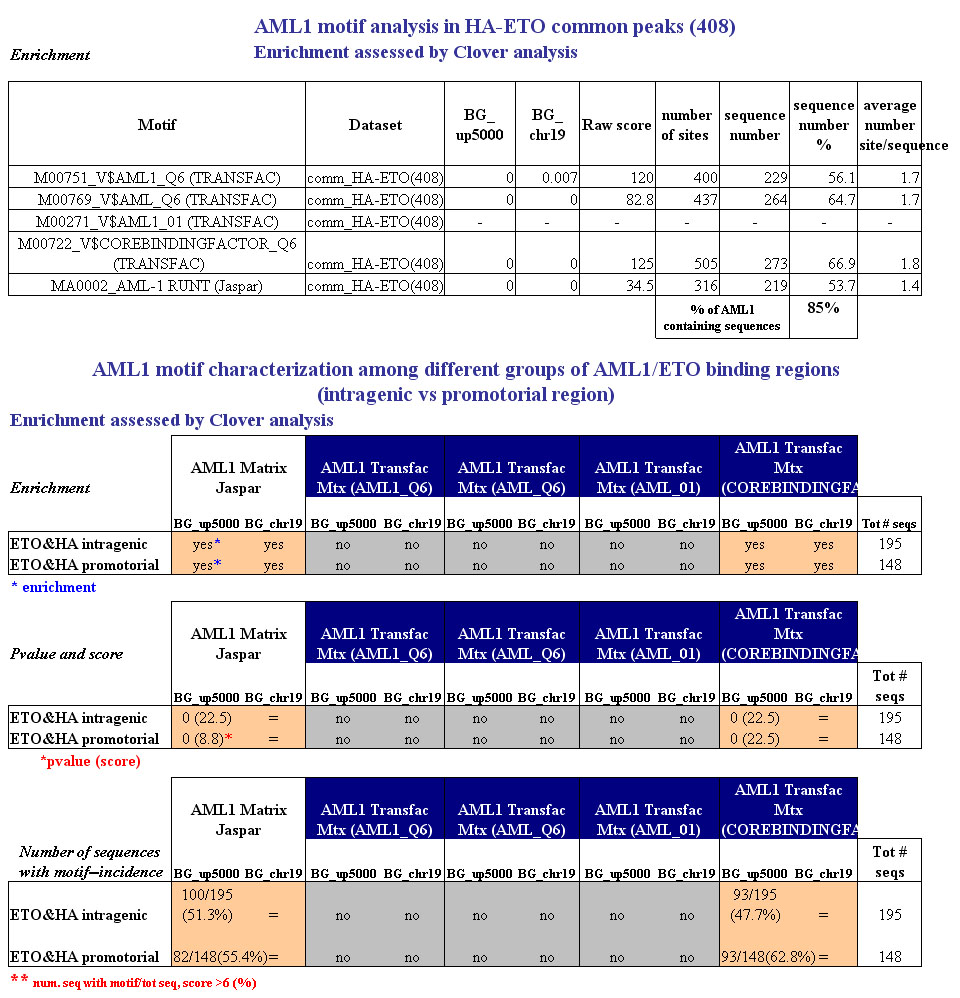


**Figure S10: Analysis of AML1 motif in the sequences corresponding to 408 AML1/ETO peaks on chromosome 19.** Upper panel: AML1/ETO binding regions were analysed for their content of AML1 consensus binding sites using all the AML1 matrices available in TRANSFAC and JASPAR databases. 85% of AML1/ETO peaks on chr. 19 contain an AML1 consensus. Lower panels: the subgroups of AML1/ETO binding regions mapping either in the promoter or within the gene body (intragenic) were analysed for their AML1 binding sites content. There is no significant difference between the two groups.

**
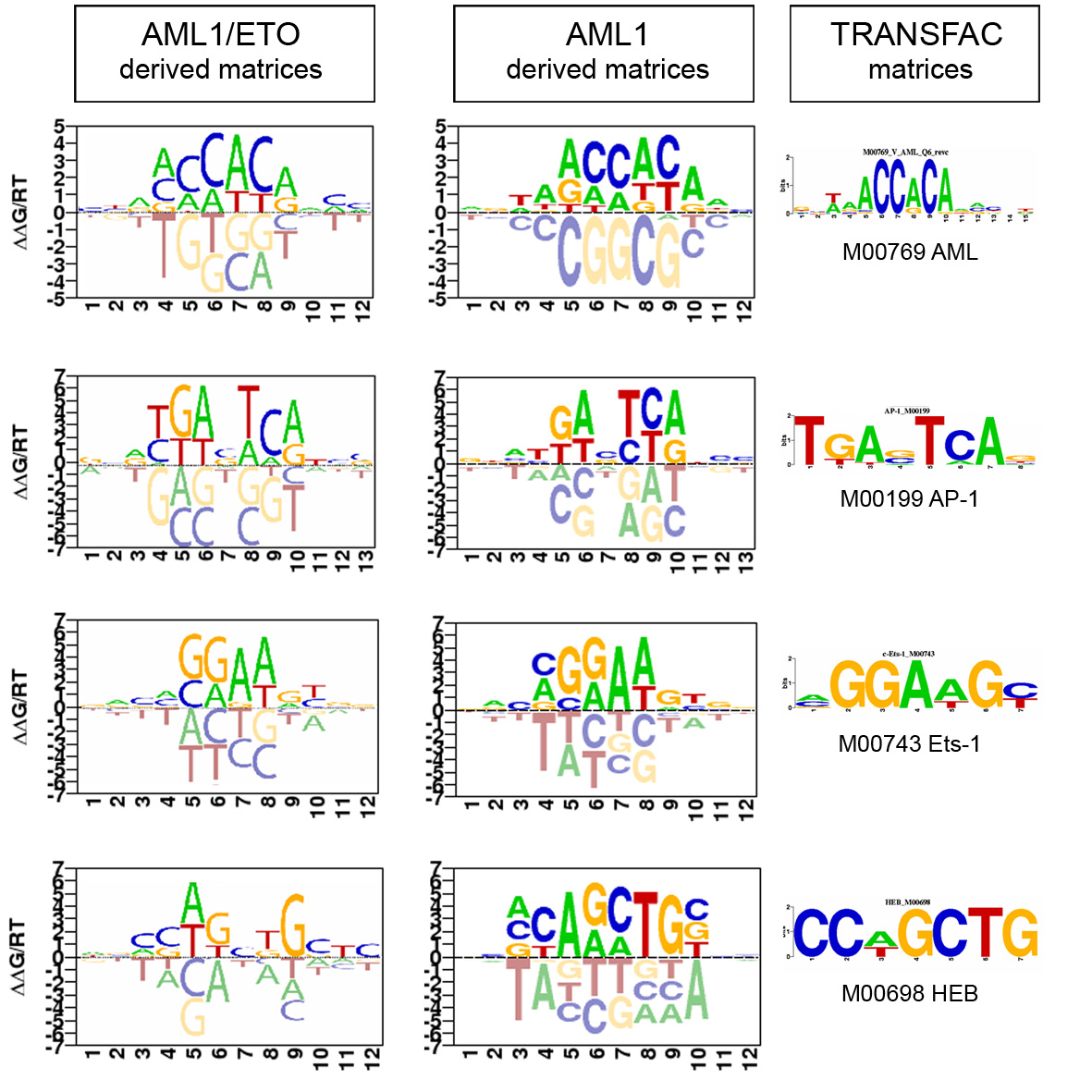
**

**Figure S11:** **Unsupervised sequence analysis of AML1/ETO and AML1 ChIP-chip data by MatrixREDUCE algorithm.** Four putative motifs were inferred from both AML1/ETO and AML1 ChIP-chip datasets from the Chr. 19 array. Motifs were aligned against TRANSFAC database to find highly significant similarities to known TFBS matrices and resulted in the identification of the AML1, AP-1, Ets-1 and HEB motifs.


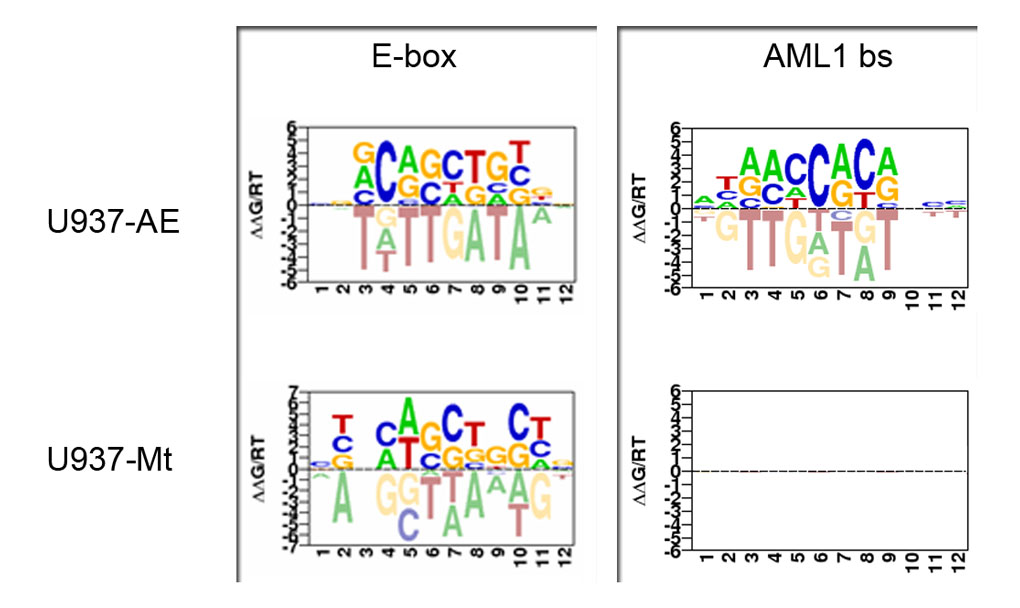


**Figure S12:** **HEB binds to regions containing an AML1 consensus only in AML1/ETO expressing cells.** MatrixREDUCE analysis infers an AML1 consensus from HEB ChIP-chip data in U937-AE but not in U937-Mt. E-box consensus is inferred in both cell lines.

**Table S11.** **Sequence analysis of AML1/ETO peaks at regulated genes.** Full list of significantly enriched matrices is reported.

| **TRANSFAC matrices enriched in DOWNREGULATED genes** | | | |
| --- | --- | --- | --- |
| **IDMtx** | **Description** | **Score** | **P-value from randomizing hg18-upstr5000** |
| M00088 | V$IK3_01 | 141 | 0 |
| M00175 | V$AP4_Q5 | 124 | 0 |
| M00698 | V$HEB_Q6 | 117 | 3.00E-03 |
| M00176 | V$AP4_Q6 | 112 | 0 |
| M00977 | V$EBF_Q6 | 97 | 5.00E-03 |
| M00962 | V$AR_Q6 | 90 | 0 |
| M00002 | V$E47_01 | 88 | 0 |
| M00470 | V$AP2GAMMA_01 | 84 | 7.00E-03 |
| M00258 | V$ISRE_01 | 80 | 5.00E-03 |
| M01043 | V$NKX25_Q5 | 78 | 0 |
| M00511 | V$ERR1_Q2 | 77 | 8.00E-03 |
| M00007 | V$ELK1_01 | 71 | 0 |
| M00960 | V$PR_Q2 | 63 | 9.00E-03 |
| M00231 | V$MEF2_02 | 60 | 1.00E-03 |
| M00155 | V$ARP1_01 | 57 | 6.00E-03 |
| M00156 | V$RORA1_01 | 53 | 0 |
| M00325 | V$NRSE_B | 51 | 0 |
| M01028 | V$NRSF_Q4 | 41 | 1.00E-03 |
| M00722 | V$COREBINDINGFACTOR_Q6 | 41 | 0 |
| M01070 | V$CMAF_01 | 39 | 4.00E-03 |
| M01022 | V$LEF1_Q2_01 | 35 | 6.00E-03 |
| M00289 | V$HFH3_01 | 24 | 1.00E-03 |
| M00328 | V$PAX8_B | 21 | 3.00E-03 |
| M00961 | V$VDR_Q6 | 19 | 9.00E-03 |
| M00054 | V$NFKAPPAB_01 | 17 | 1.00E-03 |
| M00057 | V$COMP1_01 | 16 | 0 |
| M00174 | V$AP1_Q6 | 15 | 7.00E-03 |
| M00418 | V$TGIF_01 | 15 | 4.00E-03 |
| M00924 | V$AP1_Q2_01 | 15 | 2.00E-03 |
| M00342 | V$OCT1_B | 13 | 2.00E-03 |
| M00423 | V$FOXJ2_02 | 11 | 5.00E-03 |
| M00051 | V$NFKAPPAB50_01 | 11 | 1.00E-03 |
| M00532 | V$RP58_01 | 8 | 3.00E-03 |
| M00063 | V$IRF2_01 | 7 | 0 |
| M00114 | V$TAXCREB_01 | 7 | 9.00E-03 |
| M00161 | V$OCT1_05 | 6 | 1.00E-03 |
| M00966 | V$DR3_Q4 | 5 | 2.00E-03 |
| M00416 | V$CART1_01 | 5 | 0 |
| M00526 | V$GCNF_01 | 4 | 5.00E-03 |
| M00150 | V$BRACH_01 | 3 | 0 |
| M00334 | V$DTYPEPA_B | 0 | 2.00E-03 |
| M00190 | V$CEBP_Q2 | 0 | 8.00E-03 |
| M00136 | V$OCT1_02 | 0 | 1.00E-03 |
| M00623 | V$CRX_Q4 | 0 | 3.00E-03 |
| M00292 | V$FREAC4_01 | -1 | 6.00E-03 |
| M00436 | V$IPF1_Q4 | -1 | 2.00E-03 |
| M00437 | V$CHX10_01 | -1 | 7.00E-03 |
| M00241 | V$NKX25_02 | -2 | 3.00E-03 |
| M00801 | V$CREB_Q3 | -5 | 2.00E-03 |
| M00332 | V$WHN_B | -6 | 7.00E-03 |
| **TRANSFAC matrices enriched in UPREGULATED genes** | | | |
| **IDMtx** | **Description** | **Score** | **P-value from randomizing hg18-upstr5000** |
| M00791 | V$HNF3_Q6 | 103 | 0 |
| M00006 | V$MEF2_01 | 69.5 | 0 |
| M00734 | V$CIZ_01 | 66.7 | 0 |
| M00477 | V$FOXO3_01 | 60.2 | 0 |
| M00941 | V$MEF2_Q6_01 | 45.1 | 0 |
| M00915 | V$AP2_Q6_01 | 43.6 | 0 |
| M00476 | V$FOXO4_02 | 43.6 | 0 |
| M00762 | V$DR1_Q3 | 39.9 | 0 |
| M00277 | V$LMO2COM_01 | 32.7 | 0 |
| M00160 | V$SRY_02 | 31.9 | 0 |
| M00473 | V$FOXO1_01 | 30.7 | 0 |
| M00410 | V$SOX9_B1 | 22.7 | 0 |
| M00634 | V$GCM_Q2 | 12.9 | 0 |
| M00821 | V$NRF2_Q4 | 11.9 | 0 |
| M00979 | V$PAX6_Q2 | 9.37 | 0 |
| M00631 | V$FXR_Q3 | 8.91 | 0 |
| M00665 | V$SP3_Q3 | 7.08 | 0 |
| M00192 | V$GR_Q6 | 3.58 | 0 |
| M00716 | V$ZF5_01 | 1.2 | 0 |
| M00727 | V$SF1_Q6 | 0.09 | 0 |
| M00964 | V$PXR_Q2 | -0.27 | 0 |
| M00803 | V$E2F_Q2 | -0.81 | 0 |
| M01078 | V$CETS1P54_03 | -0.91 | 0 |
| M00346 | V$GATA1_05 | -1.11 | 0 |
| M00333 | V$ZF5_B | -2.15 | 0 |
| M00228 | V$VBP_01 | -2.28 | 0 |
| M01029 | V$TFE_Q6 | -2.38 | 0 |
| M00025 | V$ELK1_02 | -2.86 | 0 |
| M00260 | V$HLF_01 | -3 | 0 |
| M00736 | V$E2F1DP1_01 | -3.19 | 0 |
| M00737 | V$E2F1DP2_01 | -3.36 | 0 |
| M00481 | V$AR_01 | -3.38 | 0 |
| M00984 | V$PEBP_Q6 | -3.43 | 0 |
| M00430 | V$E2F1_Q4 | -3.47 | 0 |
| M00285 | V$TCF11_01 | -3.57 | 0 |
| M00003 | V$VMYB_01 | -3.61 | 0 |
| M00431 | V$E2F1_Q6 | -3.81 | 0 |
| M00426 | V$E2F_Q4 | -3.85 | 0 |
| M00739 | V$E2F4DP2_01 | -3.86 | 0 |
| M00427 | V$E2F_Q6 | -3.92 | 0 |
| M00940 | V$E2F1_Q6_01 | -3.98 | 0 |
| M00326 | V$PAX1_B | -4.16 | 0 |
| M00050 | V$E2F_02 | -4.25 | 0 |
| M00425 | V$E2F_Q3 | -4.5 | 0 |
| M00034 | V$P53_01 | -4.6 | 0 |
| M01002 | V$DEAF1_02 | -4.68 | 0 |
| M00738 | V$E2F4DP1_01 | -4.74 | 0 |
| M00740 | V$E2F1DP1RB_01 | -4.82 | 0 |
| **TRANSFAC matrices commonly enriched in regulated genes** | | | |
| **IDMtx** | **Description** | **Score** | **P-value from randomizing hg18-upstr5000** |
| M00933 | V$SP1_Q2_01 | 326 | 0 |
| M00931 | V$SP1_Q6_01 | 300 | 0 |
| M00649 | V$MAZ_Q6 | 300 | 0 |
| M01066 | V$BLIMP1_Q6 | 291 | 0 |
| M00340 | V$ETS2_B | 288 | 0 |
| M00658 | V$PU1_Q6 | 282 | 0 |
| M00339 | V$ETS1_B | 268 | 0 |
| M00971 | V$ETS_Q6 | 267 | 0 |
| M00699 | V$ICSBP_Q6 | 248 | 0 |
| M00972 | V$IRF_Q6_01 | 233 | 0 |
| M00500 | V$STAT6_02 | 227 | 0 |
| M00648 | V$MAF_Q6 | 222 | 0 |
| M00380 | V$PAX4_04 | 216 | 1.00E-03 |
| M00302 | V$NFAT_Q6 | 209 | 0 |
| M00255 | V$GC_01 | 200 | 7.00E-03 |
| M00331 | V$TAACC_B | 196 | 0 |
| M00932 | V$SP1_Q4_01 | 193 | 3.00E-03 |
| M00777 | V$STAT_Q6 | 183 | 0 |
| M00196 | V$SP1_Q6 | 176 | 5.00E-03 |
| M00935 | V$NFAT_Q4_01 | 171 | 0 |
| M00531 | V$NERF_Q2 | 171 | 0 |
| M00083 | V$MZF1_01 | 170 | 0 |
| M00499 | V$STAT5A_04 | 162 | 0 |
| M00491 | V$MAZR_01 | 160 | 0 |
| M00746 | V$ELF1_Q6 | 153 | 0 |
| M00747 | V$IRF1_Q6 | 149 | 0 |
| M00678 | V$TEL2_Q6 | 145 | 0 |
| M00772 | V$IRF_Q6 | 145 | 0 |
| M00657 | V$PTF1BETA_Q6 | 144 | 0 |
| M00774 | V$NFKB_Q6_01 | 141 | 0 |
| M00257 | V$RREB1_01 | 136 | 2.00E-03 |
| M00982 | V$KROX_Q6 | 135 | 2.00E-03 |
| M00743 | V$CETS168_Q6 | 129 | 0 |
| M01007 | V$SRF_Q5_02 | 128 | 0 |
| M00695 | V$ETF_Q6 | 127 | 1.00E-03 |
| M01068 | V$UF1H3BETA_Q6 | 127 | 0 |
| M00644 | V$LBP1_Q6 | 124 | 0 |
| M00999 | V$AIRE_01 | 121 | 0 |
| M01004 | V$HELIOSA_02 | 119 | 0 |
| M00632 | V$GATA4_Q3 | 117 | 0 |
| M00493 | V$STAT5A_03 | 109 | 0 |
| M00459 | V$STAT5B_01 | 101 | 0 |
| M00377 | V$PAX4_02 | 101 | 6.00E-03 |
| M00498 | V$STAT4_01 | 99 | 0 |
| M01012 | V$HNF3_Q6_01 | 99 | 0 |
| M00655 | V$PEA3_Q6 | 98 | 0 |
| M00406 | V$HMEF2_Q6 | 98 | 9.00E-03 |
| M00148 | V$SRY_01 | 96 | 0 |
| M00930 | V$OCT1_Q5_01 | 95 | 0 |
| M00086 | V$IK1_01 | 94 | 0 |
| M00807 | V$EGR_Q6 | 94 | 0.01 |
| M00486 | V$PAX2_02 | 92 | 0 |
| M00250 | V$GFI1_01 | 89 | 0 |
| M00771 | V$ETS_Q4 | 88 | 0 |
| M00422 | V$FOXJ2_01 | 86 | 3.00E-03 |
| M00497 | V$STAT3_02 | 85 | 0 |
| M00496 | V$STAT1_03 | 83 | 0 |
| M00795 | V$OCT_Q6 | 82 | 0 |
| M00494 | V$STAT6_01 | 80 | 0 |
| M00056 | V$MYOGNF1_01 | 78 | 1.00E-03 |
| M00456 | V$FAC1_01 | 76 | 0 |
| M01010 | V$HMGIY_Q3 | 76 | 0 |
| M00980 | V$TBP_Q6 | 74 | 0 |
| M00489 | V$NKX62_Q2 | 74 | 0 |
| M00472 | V$FOXO4_01 | 73 | 7.00E-03 |
| M01023 | V$HSF1_Q6 | 73 | 0 |
| M00144 | V$PAX5_02 | 73 | 0 |
| M00729 | V$CDX2_Q5 | 71 | 0 |
| M00750 | V$HMGIY_Q6 | 71 | 0 |
| M01014 | V$SOX_Q6 | 69 | 0.01 |
| M00238 | V$BARBIE_01 | 68 | 0 |
| M00232 | V$MEF2_03 | 68 | 0 |
| M00912 | V$CEBP_Q2_01 | 67 | 0 |
| M00424 | V$NKX61_01 | 66 | 1.00E-03 |
| M00622 | V$CEBPGAMMA_Q6 | 64 | 0 |
| M00224 | V$STAT1_01 | 64 | 0 |
| M00081 | V$EVI1_04 | 63 | 0 |
| M00216 | V$TATA_C | 63 | 0 |
| M00800 | V$AP2_Q3 | 63 | 0 |
| M00986 | V$CHCH_01 | 61 | 0 |
| M00341 | V$GABP_B | 60 | 0 |
| M00725 | V$HP1SITEFACTOR_Q6 | 59 | 0 |
| M00992 | V$FOXP3_Q4 | 58 | 0 |
| M00194 | V$NFKB_Q6 | 57 | 0 |
| M00457 | V$STAT5A_01 | 57 | 0 |
| M00074 | V$CETS1P54_02 | 56 | 0 |
| M00415 | V$AREB6_04 | 53 | 0 |
| M00097 | V$PAX6_01 | 53 | 0 |
| M00108 | V$NRF2_01 | 52 | 0 |
| M00138 | V$OCT1_04 | 50 | 0 |
| M00538 | V$HTF_01 | 49 | 0 |
| M00802 | V$PIT1_Q6 | 49 | 0 |
| M00453 | V$IRF7_01 | 49 | 0 |
| M00671 | V$TCF4_Q5 | 47 | 0 |
| M00268 | V$XFD2_01 | 46 | 0 |
| M00318 | V$LPOLYA_B | 46 | 4.00E-03 |
| M00225 | V$STAT3_01 | 42 | 0 |
| M00099 | V$S8_01 | 41 | 0 |
| M00189 | V$AP2_Q6 | 39 | 1.00E-03 |
| M00641 | V$HSF_Q6 | 39 | 0 |
| M00638 | V$HNF4ALPHA_Q6 | 38 | 0 |
| M00032 | V$CETS1P54_01 | 36 | 0 |
| M00212 | V$POLY_C | 36 | 0 |
| M00672 | V$TEF_Q6 | 34 | 0 |
| M00162 | V$OCT1_06 | 32 | 0 |
| M01011 | V$HNF1_Q6_01 | 31 | 0 |
| M00096 | V$PBX1_01 | 31 | 0 |
| M00100 | V$CDXA_01 | 31 | 0 |
| M00471 | V$TBP_01 | 31 | 0 |
| M00447 | V$AR_Q2 | 29 | 0 |
| M00210 | V$OCT_C | 28 | 0 |
| M01075 | V$PLZF_02 | 28 | 0 |
| M00492 | V$STAT1_02 | 28 | 0 |
| M00267 | V$XFD1_01 | 28 | 0 |
| M00405 | V$MMEF2_Q6 | 27 | 0 |
| M00991 | V$CDX_Q5 | 27 | 0 |
| M00460 | V$STAT5A_02 | 26 | 0 |
| M00252 | V$TATA_01 | 26 | 0 |
| M00320 | V$MTATA_B | 25 | 0 |
| M00639 | V$HNF6_Q6 | 25 | 1.00E-03 |
| M00208 | V$NFKB_C | 23 | 0 |
| M00293 | V$FREAC7_01 | 22 | 0 |
| M00101 | V$CDXA_02 | 22 | 0 |
| M00403 | V$AMEF2_Q6 | 21 | 0 |
| M00223 | V$STAT_01 | 16 | 0 |
| M00465 | V$POU6F1_01 | 12 | 0 |
| M00407 | V$RSRFC4_Q2 | 11 | 0 |
| M00058 | V$HEN1_02 | 11 | 2.00E-03 |
| M00133 | V$TST1_01 | 10 | 0 |
| M00131 | V$HNF3B_01 | 10 | 0 |
| M00269 | V$XFD3_01 | 9 | 0 |
| M00790 | V$HNF1_Q6 | 7 | 0 |
| M00026 | V$RSRFC4_01 | 6 | 2.00E-03 |
| M00062 | V$IRF1_01 | 5 | 6.00E-03 |
| M00129 | V$HFH1_01 | 3 | 6.00E-03 |
| M00616 | V$AFP1_Q6 | 2 | 1.00E-03 |
| M00707 | V$TFIIA_Q6 | 2 | 0 |
| M00146 | V$HSF1_01 | 1 | 1.00E-03 |
| M00394 | V$MSX1_01 | 1 | 4.00E-03 |
| M00132 | V$HNF1_01 | 1 | 1.00E-03 |
| M01000 | V$AIRE_02 | 1 | 0 |
| M00206 | V$HNF1_C | 0 | 1.00E-03 |
| M00310 | V$APOLYA_B | 0 | 0 |
| M00116 | V$CEBPA_01 | 0 | 8.00E-03 |
| M00147 | V$HSF2_01 | 0 | 5.00E-03 |
| M00445 | V$XVENT1_01 | 0 | 0.01 |
| M00621 | V$CEBPDELTA_Q6 | -1 | 3.00E-03 |
| M00478 | V$CDC5_01 | -2 | 3.00E-03 |
| M00227 | V$VMYB_02 | -6 | 9.00E-03 |
